# Supplementary figures and images for: RNA-binding deficient TDP-43 drives cognitive decline in a mouse model of TDP-43 proteinopathy
Source: eLife. 2023 Oct 11;12:RP85921. doi: 10.7554/eLife.85921 (PMC10567115; doi:10.7554/eLife.85921)

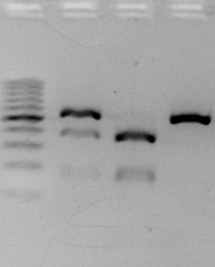

Supplement: Figure 2—figure supplement 1—source data 1. [file elife-85921-fig2-figsupp1-data1.zip › Fig 2 Supp Fig 1 Source Data/Fig2_SuppFig1_SourceData1_A.tif]

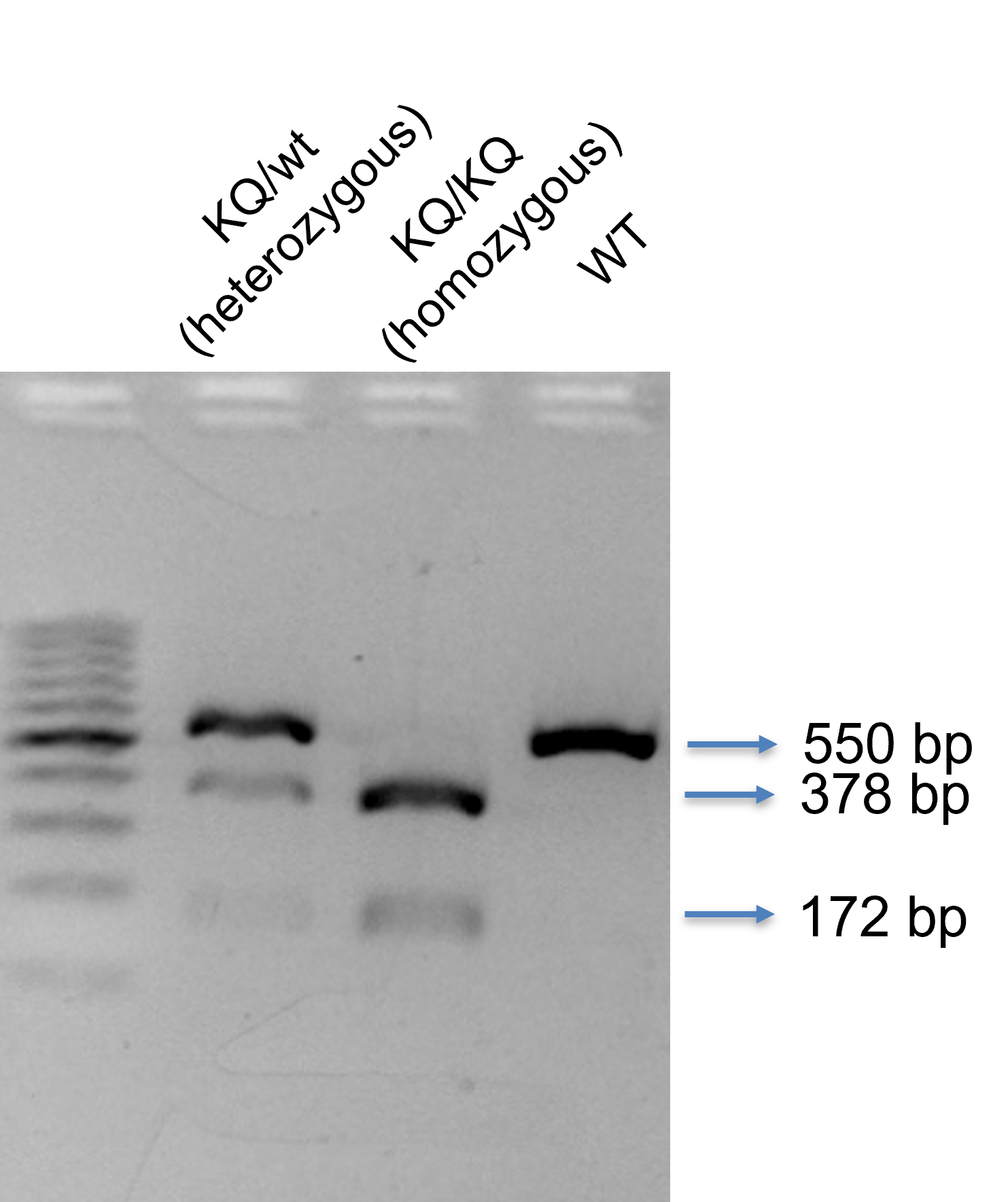

Supplement: Figure 2—figure supplement 1—source data 1. [file elife-85921-fig2-figsupp1-data1.zip › Fig 2 Supp Fig 1 Source Data/Fig2_SuppFig1_SourceData1_B.png]

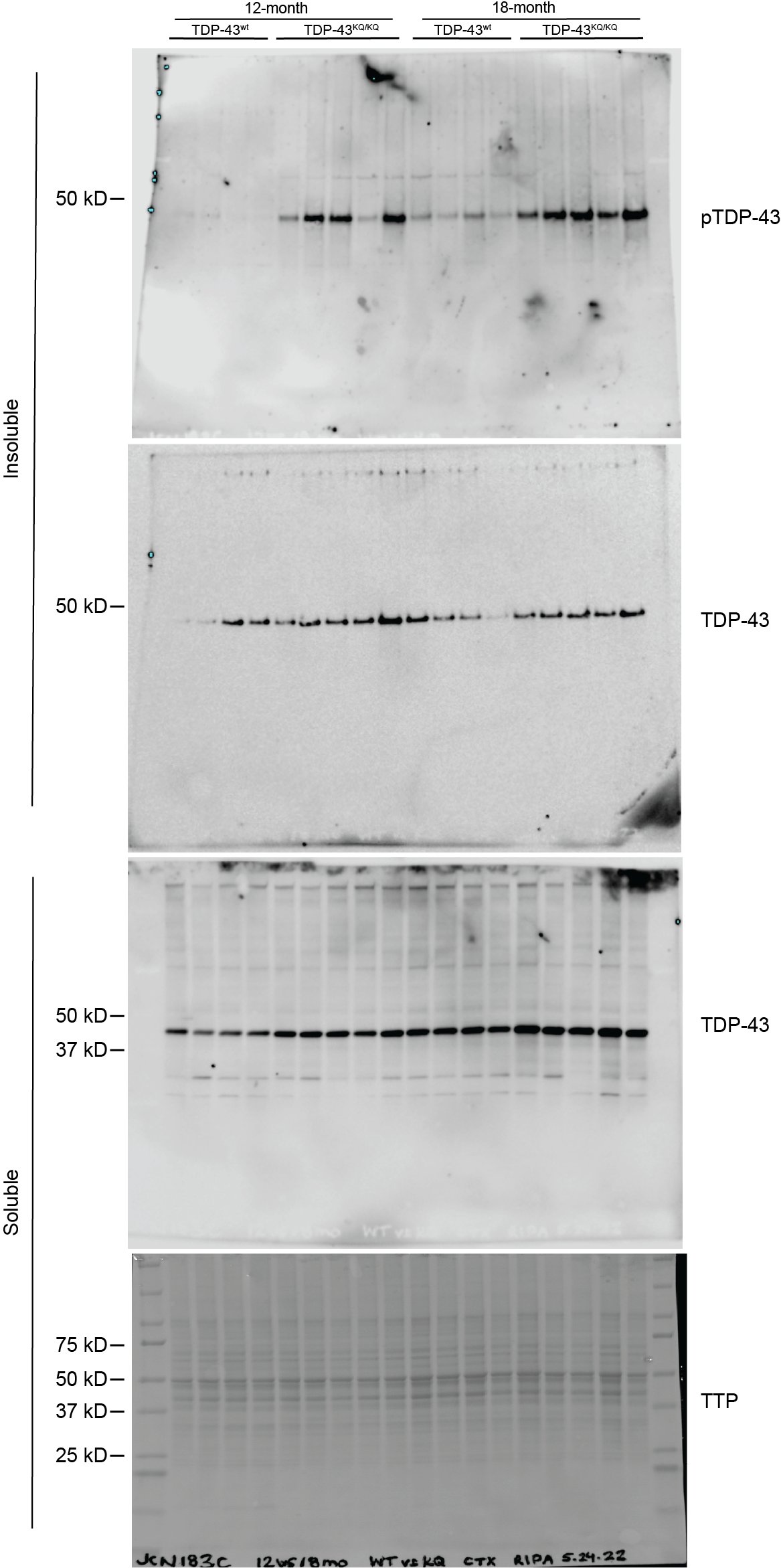

Supplement: Figure 6—source data 2. [file elife-85921-fig6-data2.zip › Fig6_SourceData2/Fig6_SourceData2_A.png]

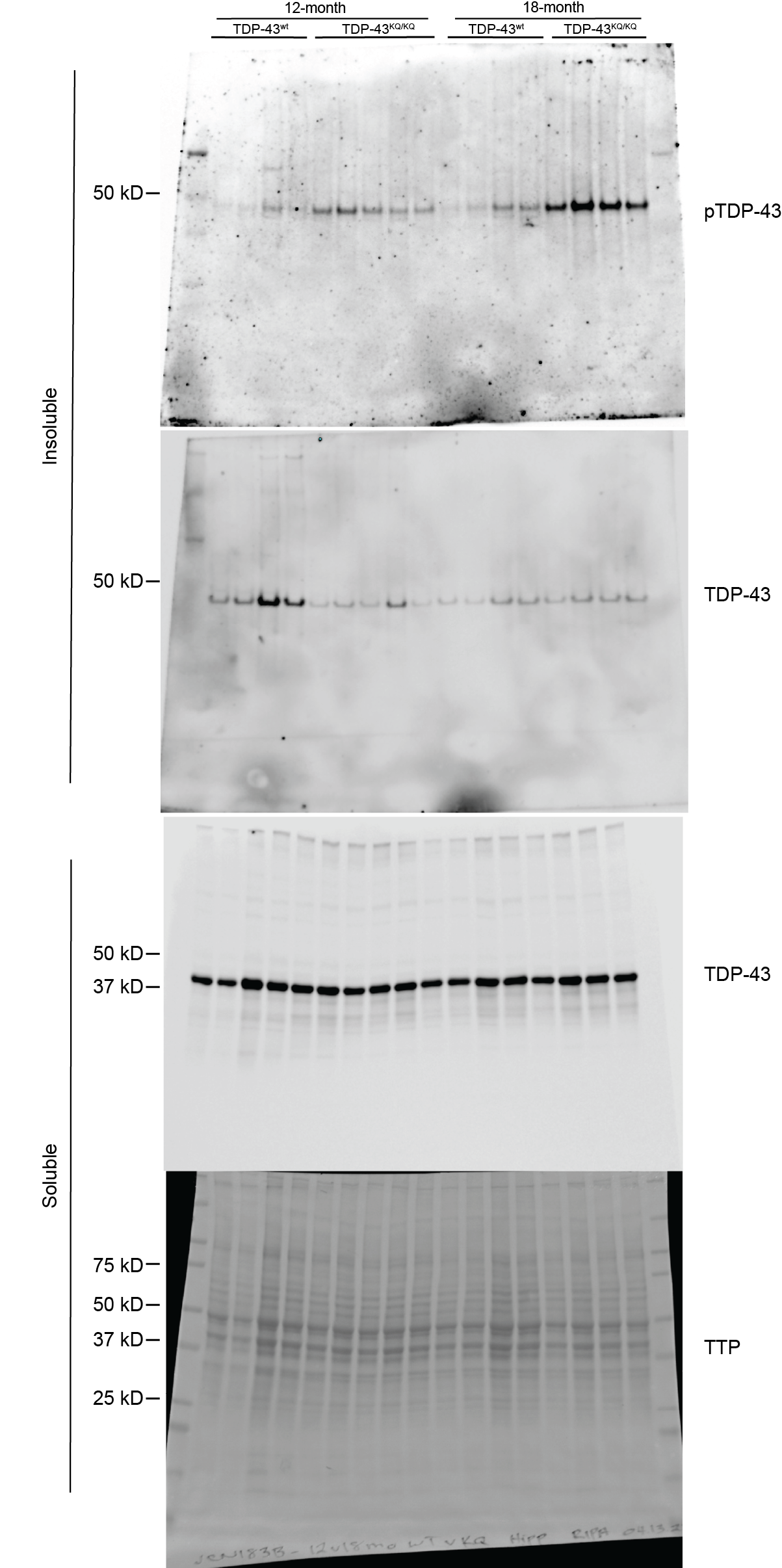

Supplement: Figure 6—source data 2. [file elife-85921-fig6-data2.zip › Fig6_SourceData2/Fig6_SourceData2_D.png]

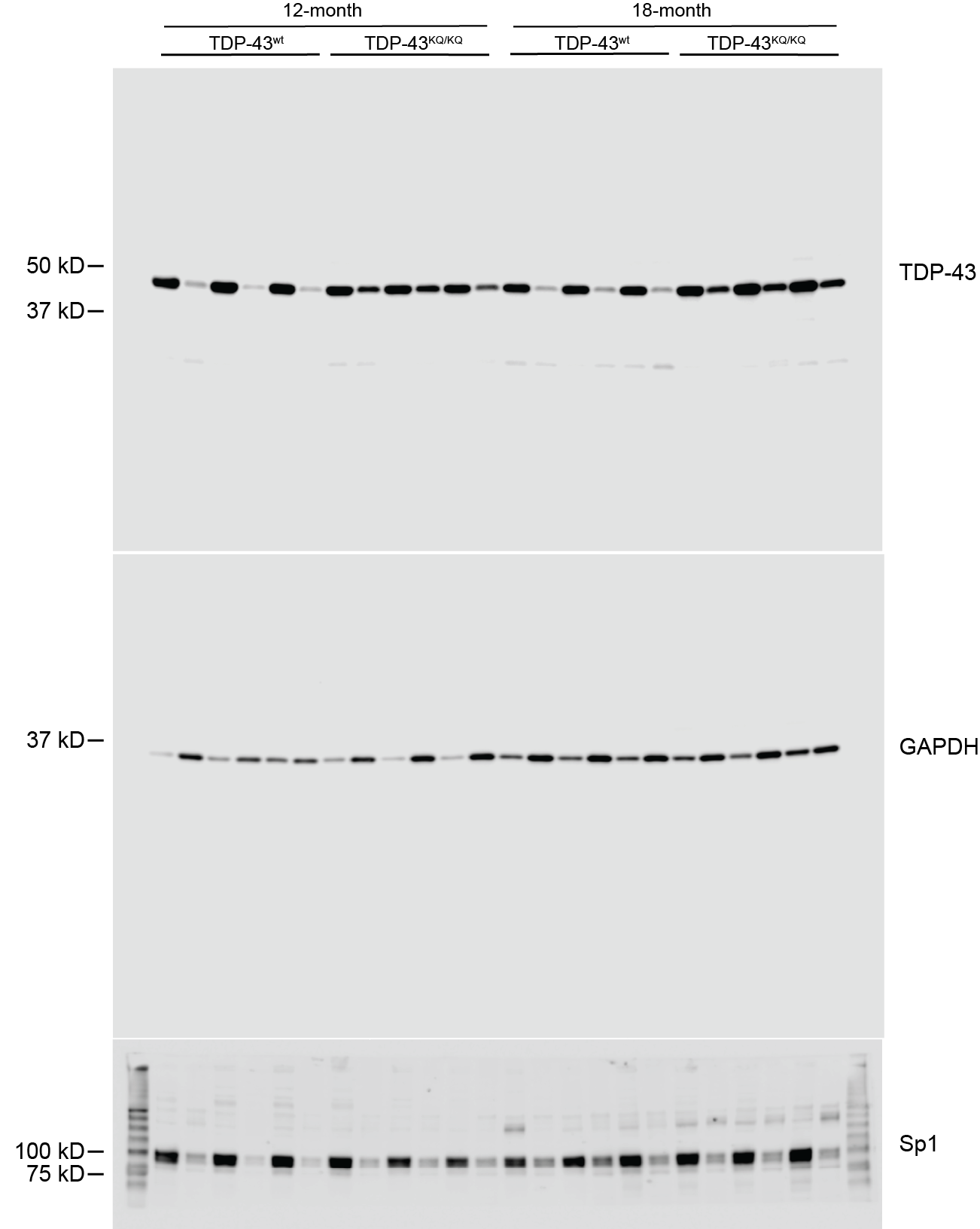

Supplement: Figure 6—source data 2. [file elife-85921-fig6-data2.zip › Fig6_SourceData2/Fig6_SourceData2_G.png]

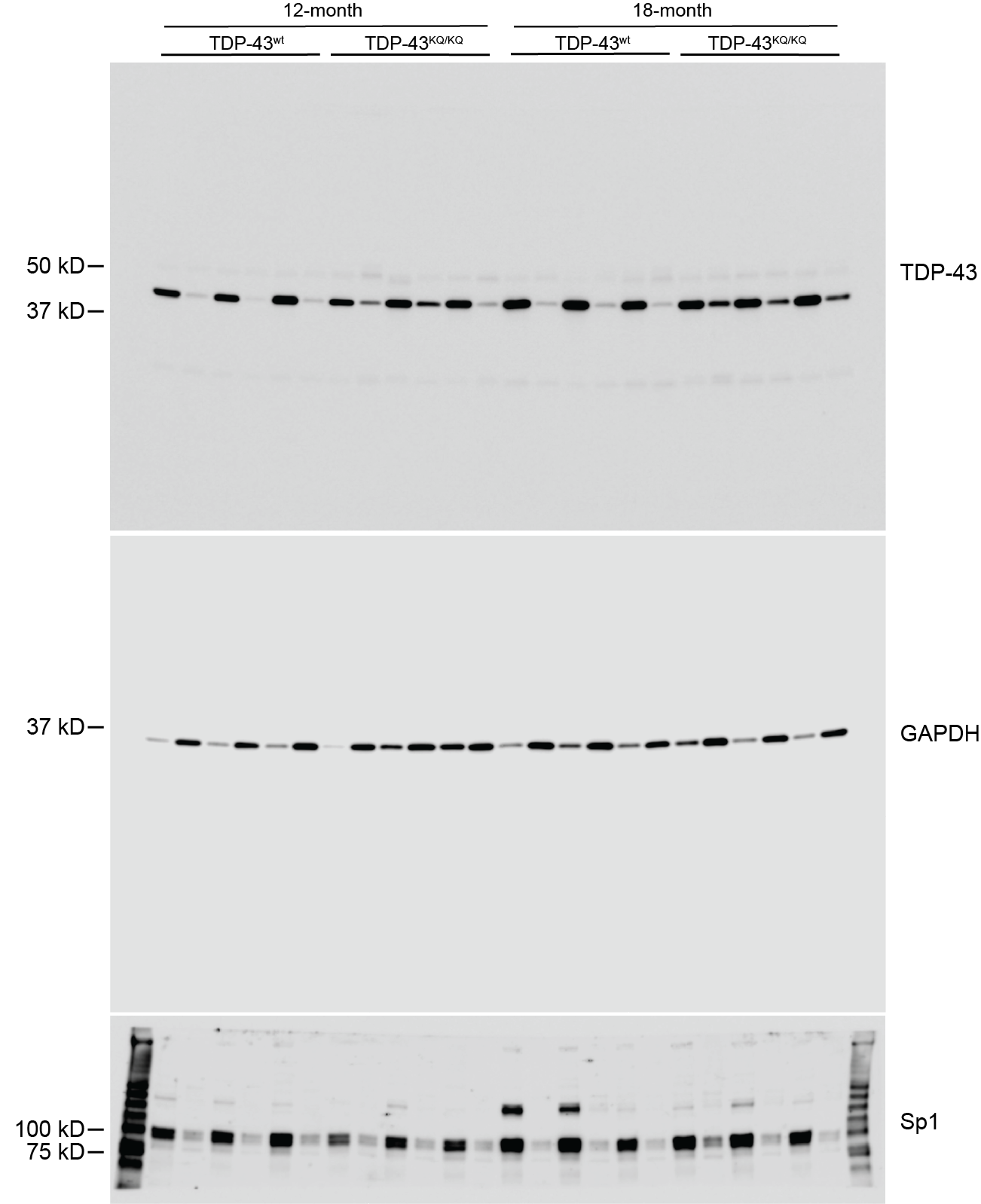

Supplement: Figure 6—source data 2. [file elife-85921-fig6-data2.zip › Fig6_SourceData2/Fig6_SourceData2_J.png]

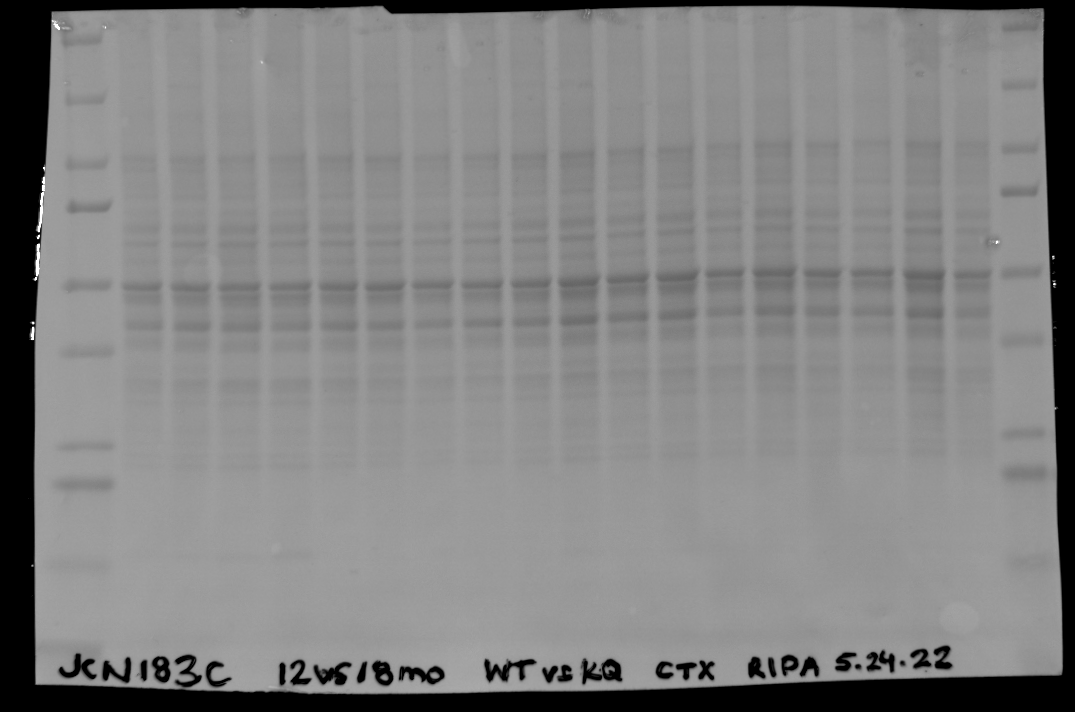

Supplement: Figure 6—source data 3. [file elife-85921-fig6-data3.zip › Fig6_SourceData3/Fig6A_CTX_RIPAsol_Ponceau-TTP_sourceblot.tif]

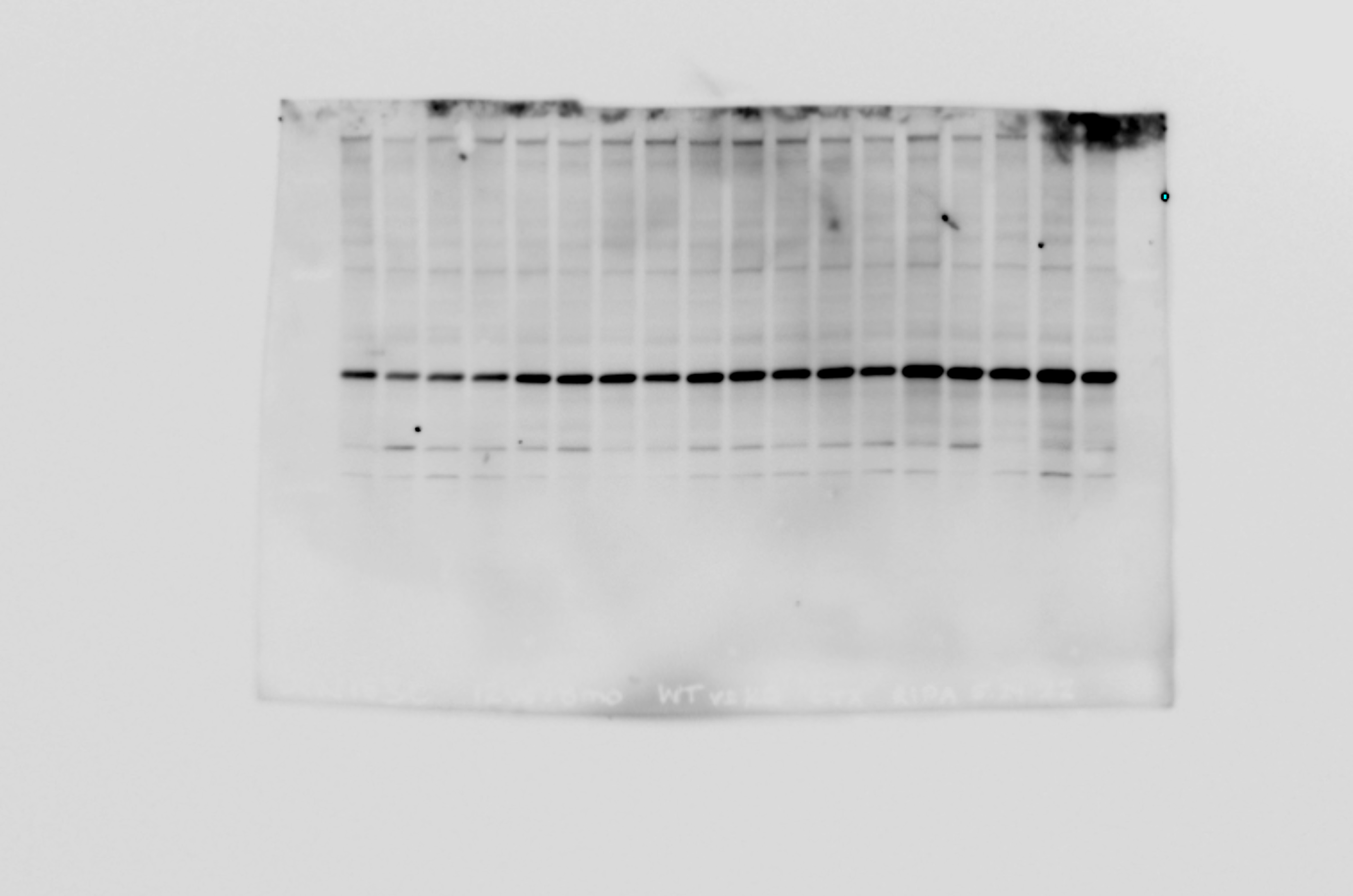

Supplement: Figure 6—source data 3. [file elife-85921-fig6-data3.zip › Fig6_SourceData3/Fig6A_CTX_RIPAsol_TDP43_sourceblot.tif]

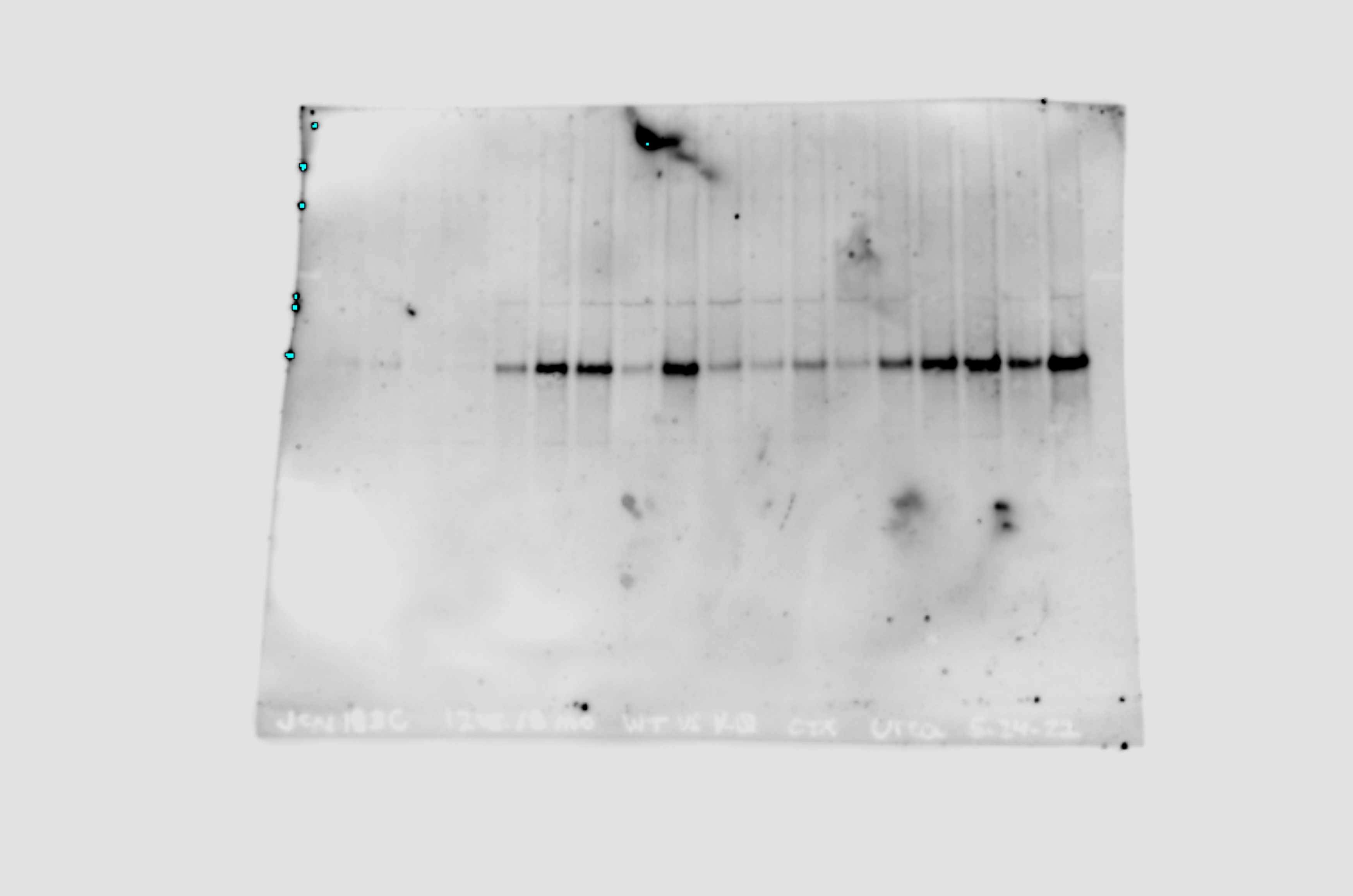

Supplement: Figure 6—source data 3. [file elife-85921-fig6-data3.zip › Fig6_SourceData3/Fig6A_CTX_Urea-Insol_pTDP43_sourceblot.tif]

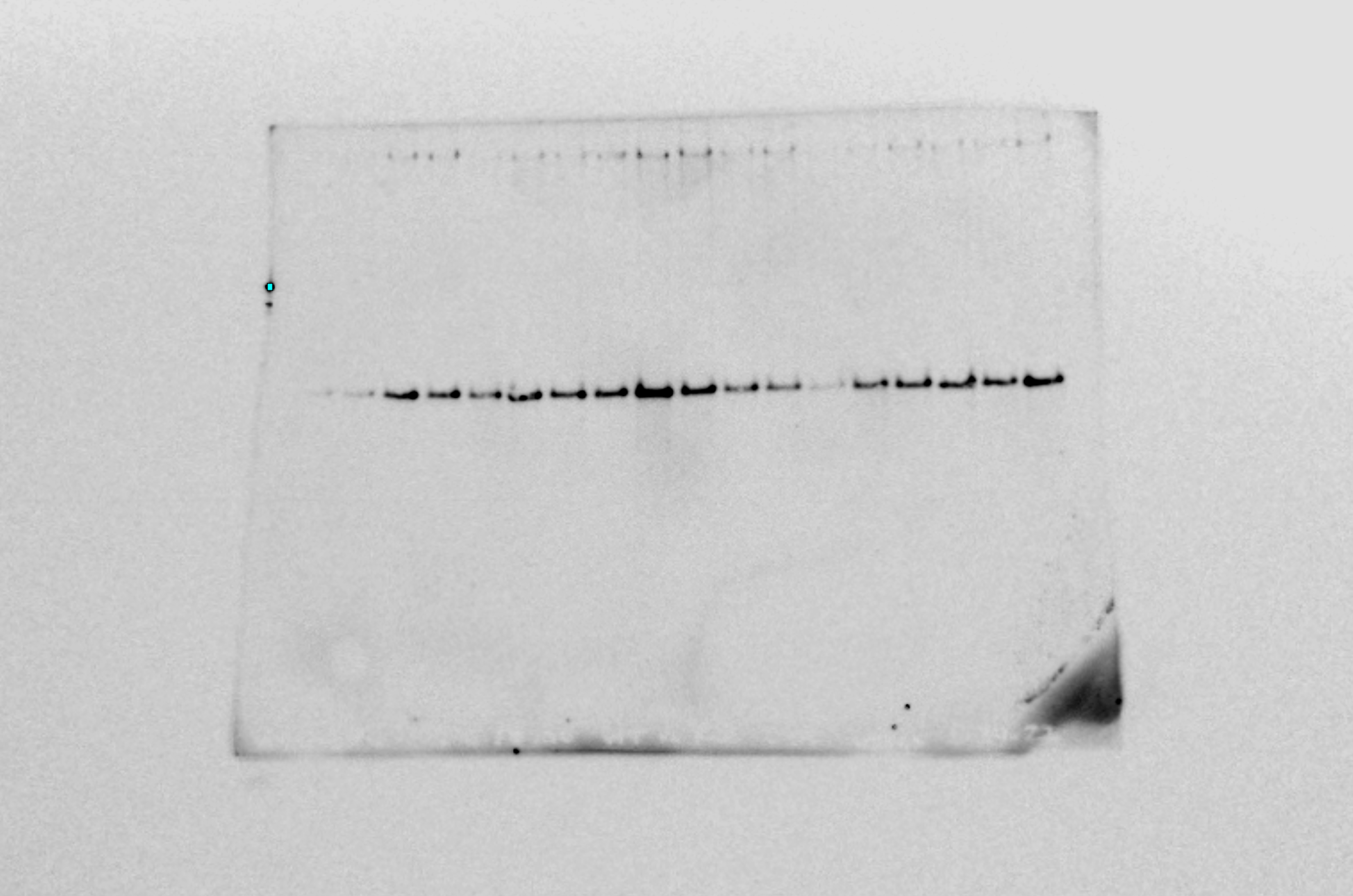

Supplement: Figure 6—source data 3. [file elife-85921-fig6-data3.zip › Fig6_SourceData3/Fig6A_CTX_Urea-Insol_TDP43_sourceblot.tif]

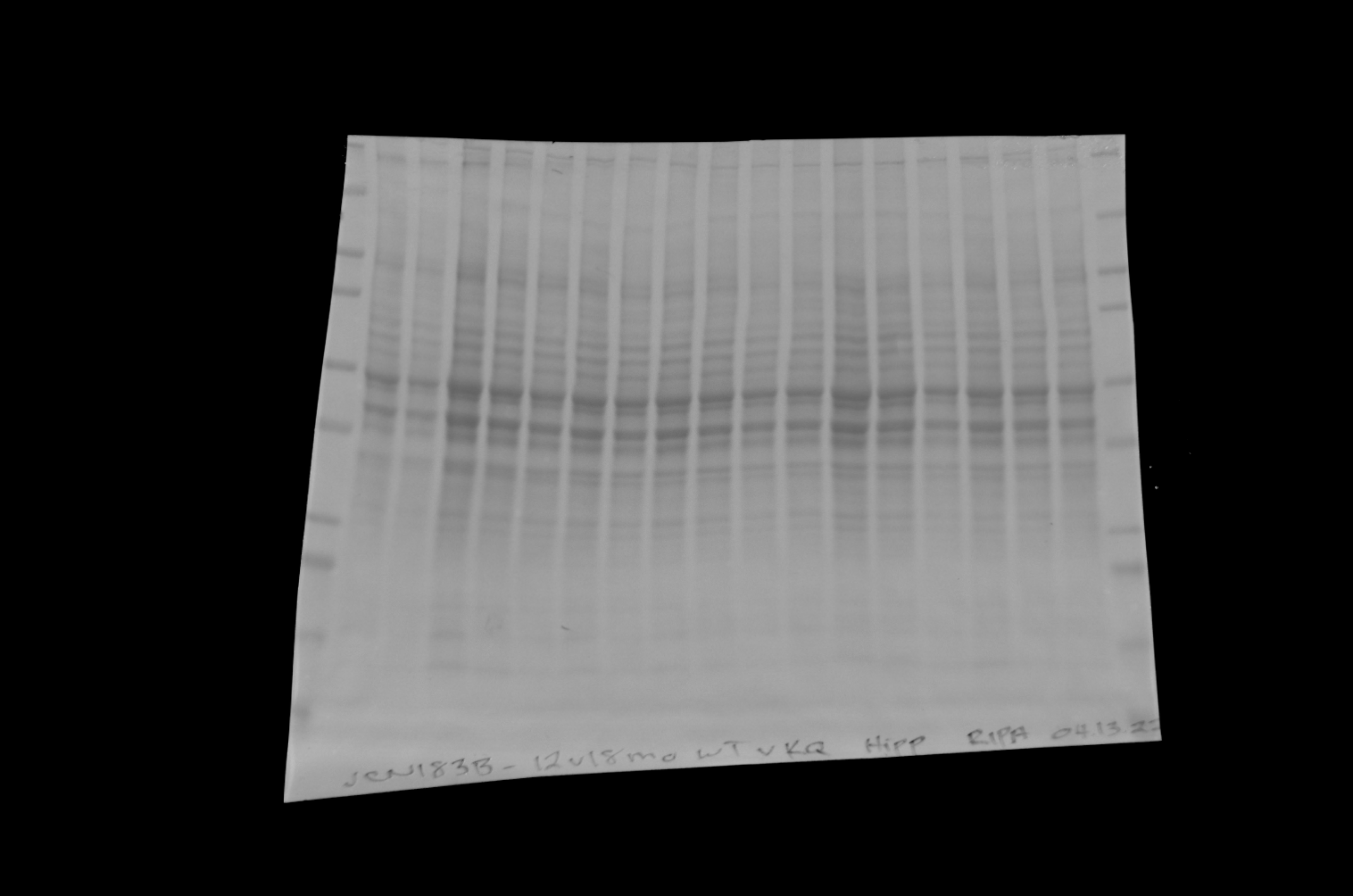

Supplement: Figure 6—source data 3. [file elife-85921-fig6-data3.zip › Fig6_SourceData3/Fig6D_Hipp_RIPAsol_Ponceau-TTP_sourceblot.tif]

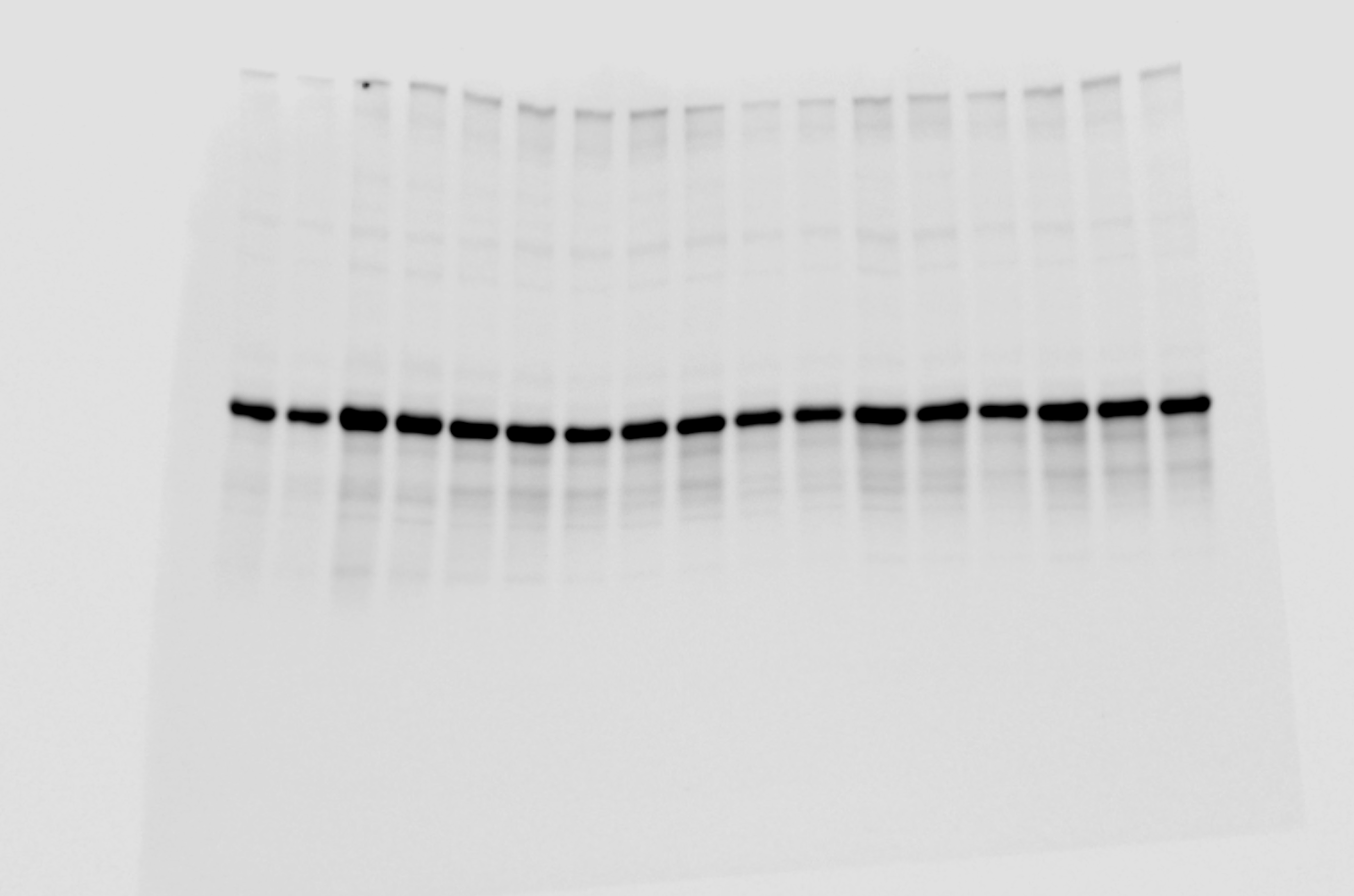

Supplement: Figure 6—source data 3. [file elife-85921-fig6-data3.zip › Fig6_SourceData3/Fig6D_Hipp_RIPAsolTDP43_sourceblot.tif]

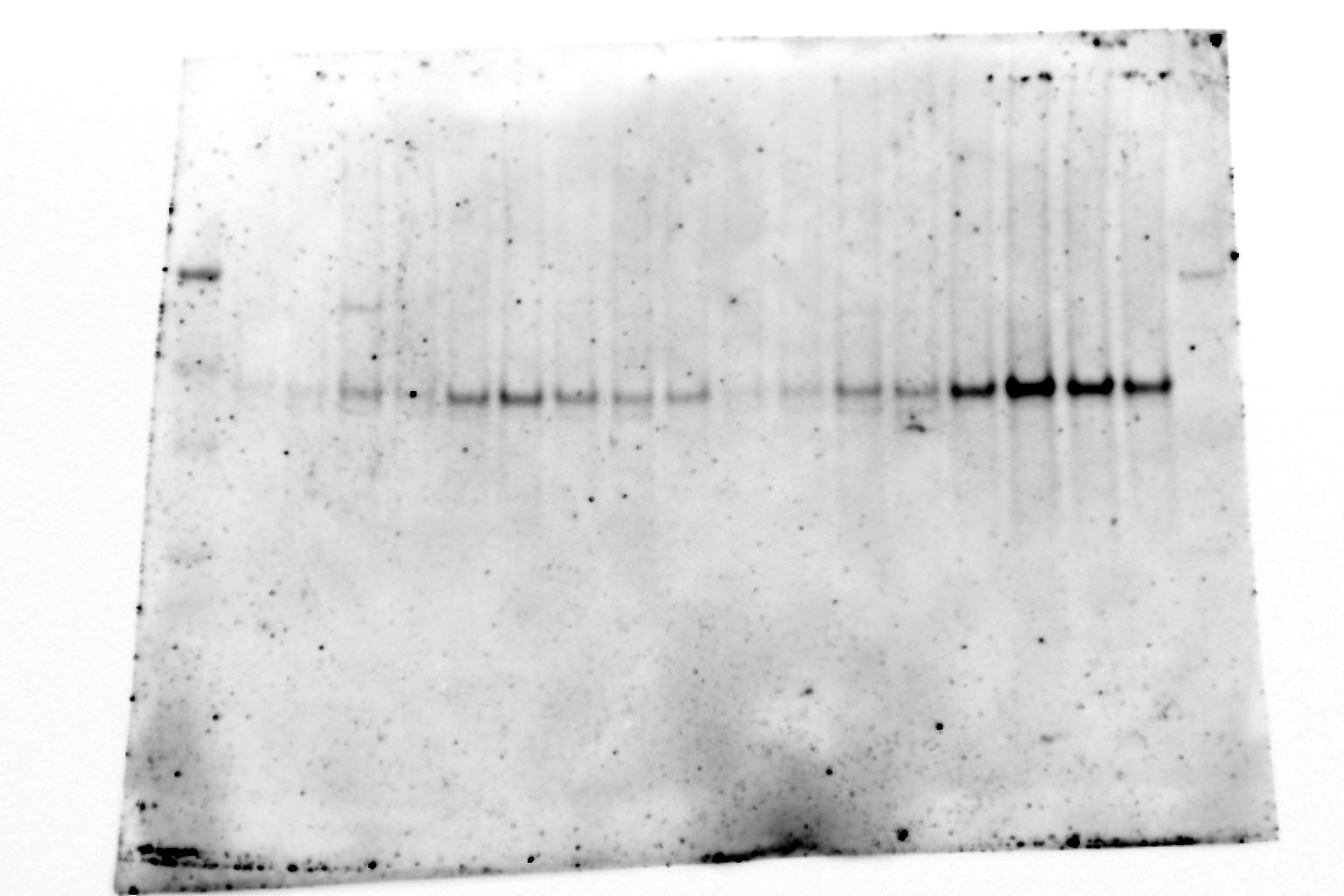

Supplement: Figure 6—source data 3. [file elife-85921-fig6-data3.zip › Fig6_SourceData3/Fig6D_Hipp_Urea-Insol_pTDP43_sourceblot.tif]

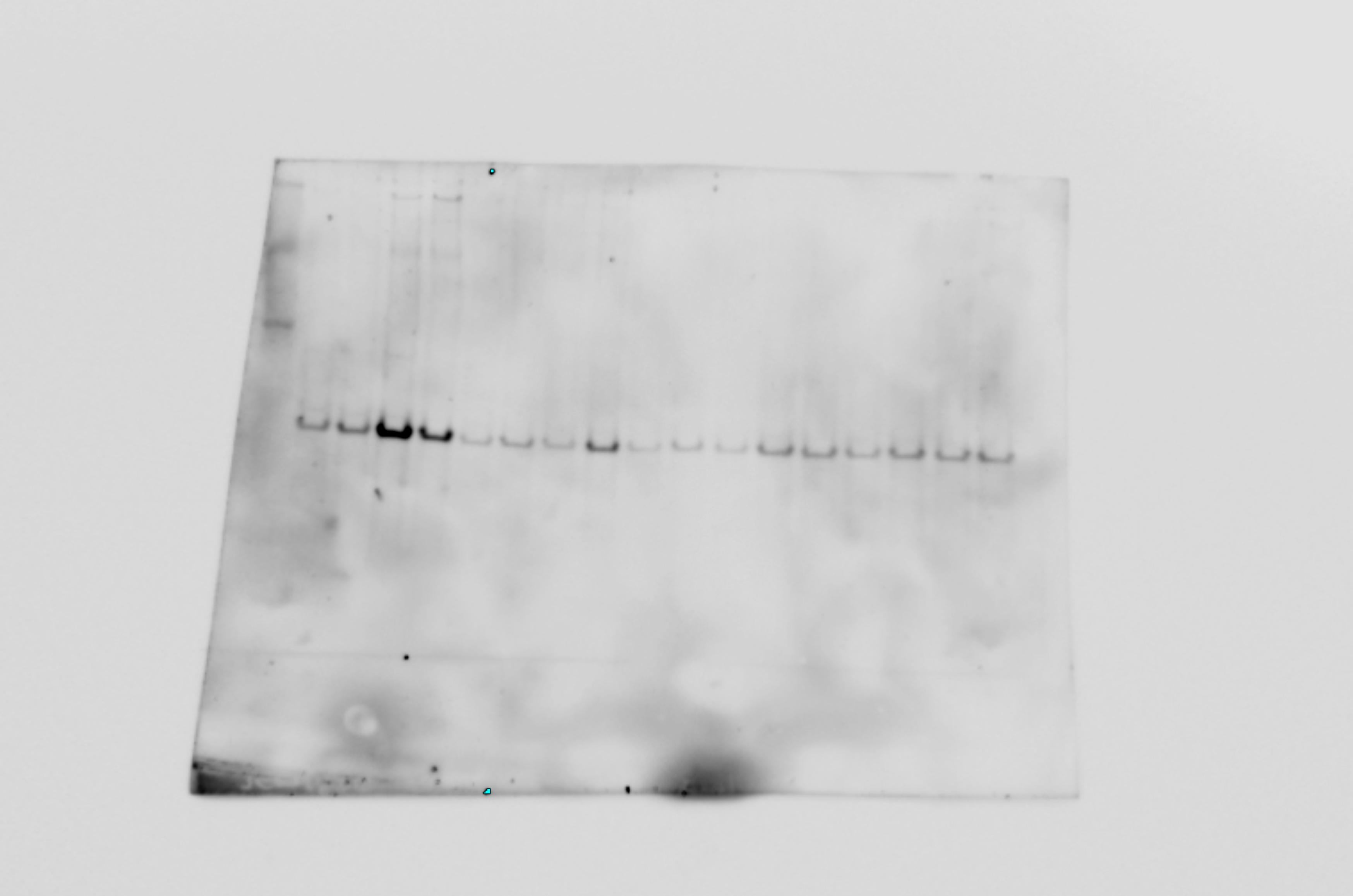

Supplement: Figure 6—source data 3. [file elife-85921-fig6-data3.zip › Fig6_SourceData3/Fig6D_Hipp_Urea-Insol_TDP43_sourceblot.tif]

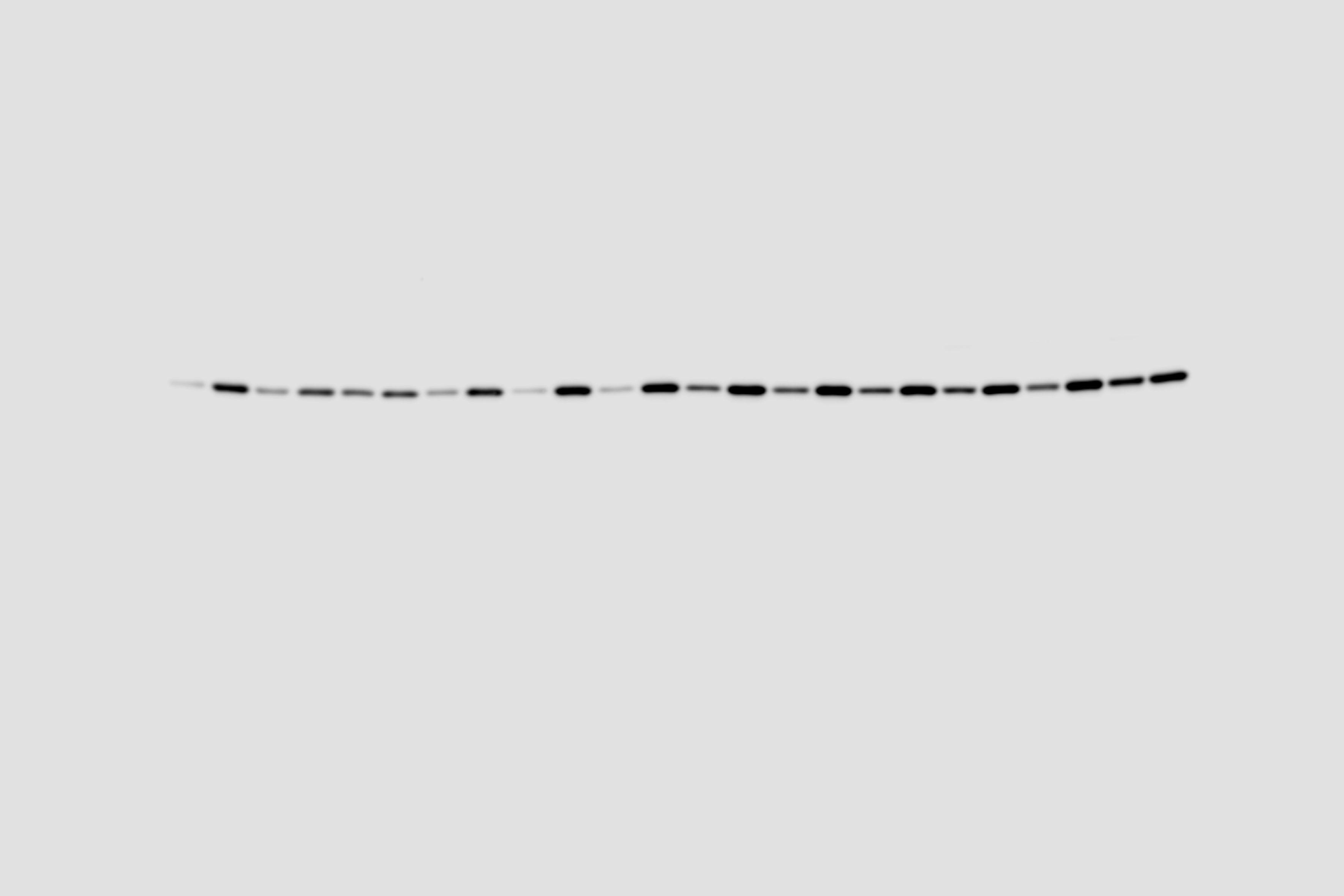

Supplement: Figure 6—source data 3. [file elife-85921-fig6-data3.zip › Fig6_SourceData3/Fig6G_CTX_GAPDH_sourceblot.tif]

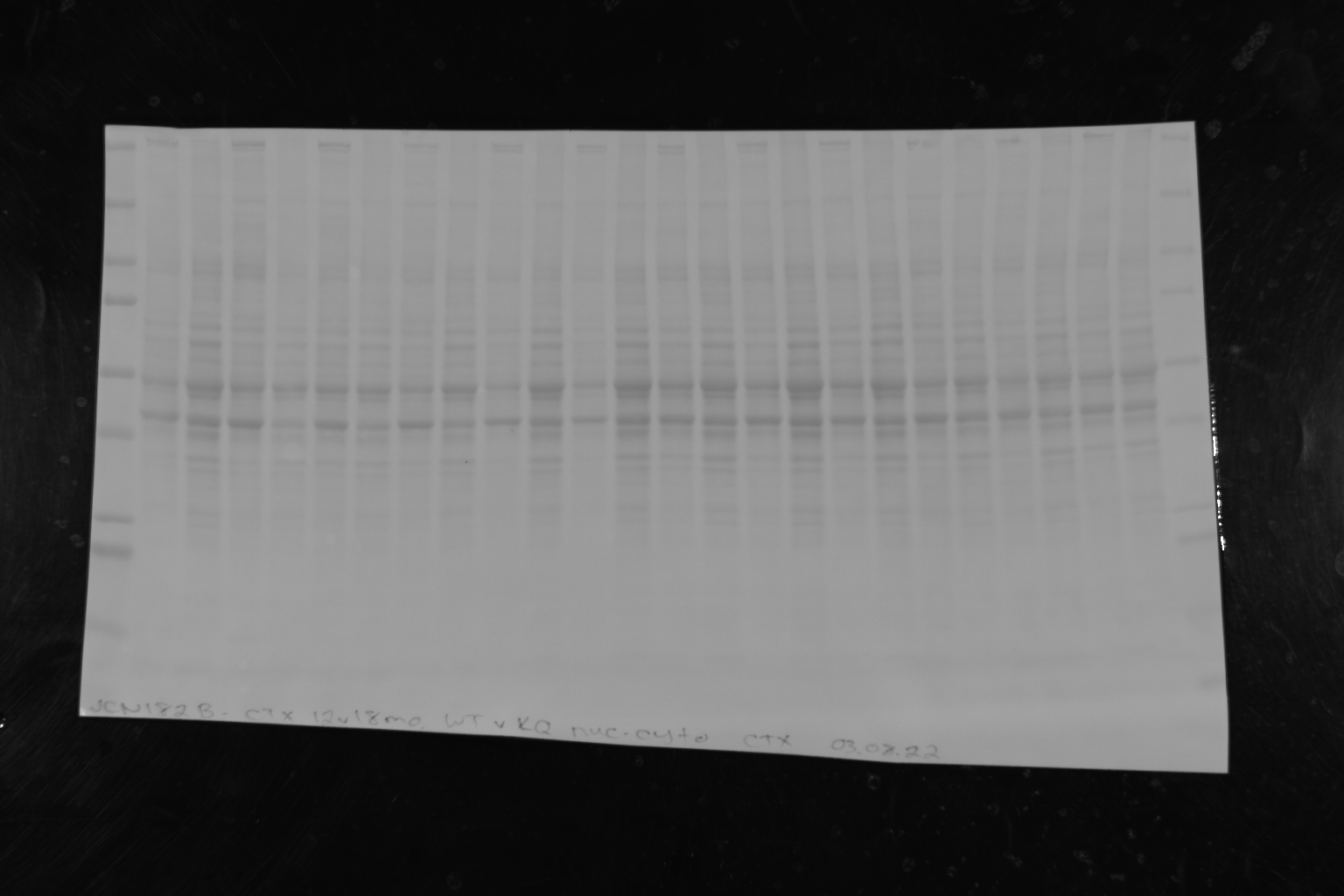

Supplement: Figure 6—source data 3. [file elife-85921-fig6-data3.zip › Fig6_SourceData3/Fig6G_CTX_Ponceau-TTP_sourceblot.tif]

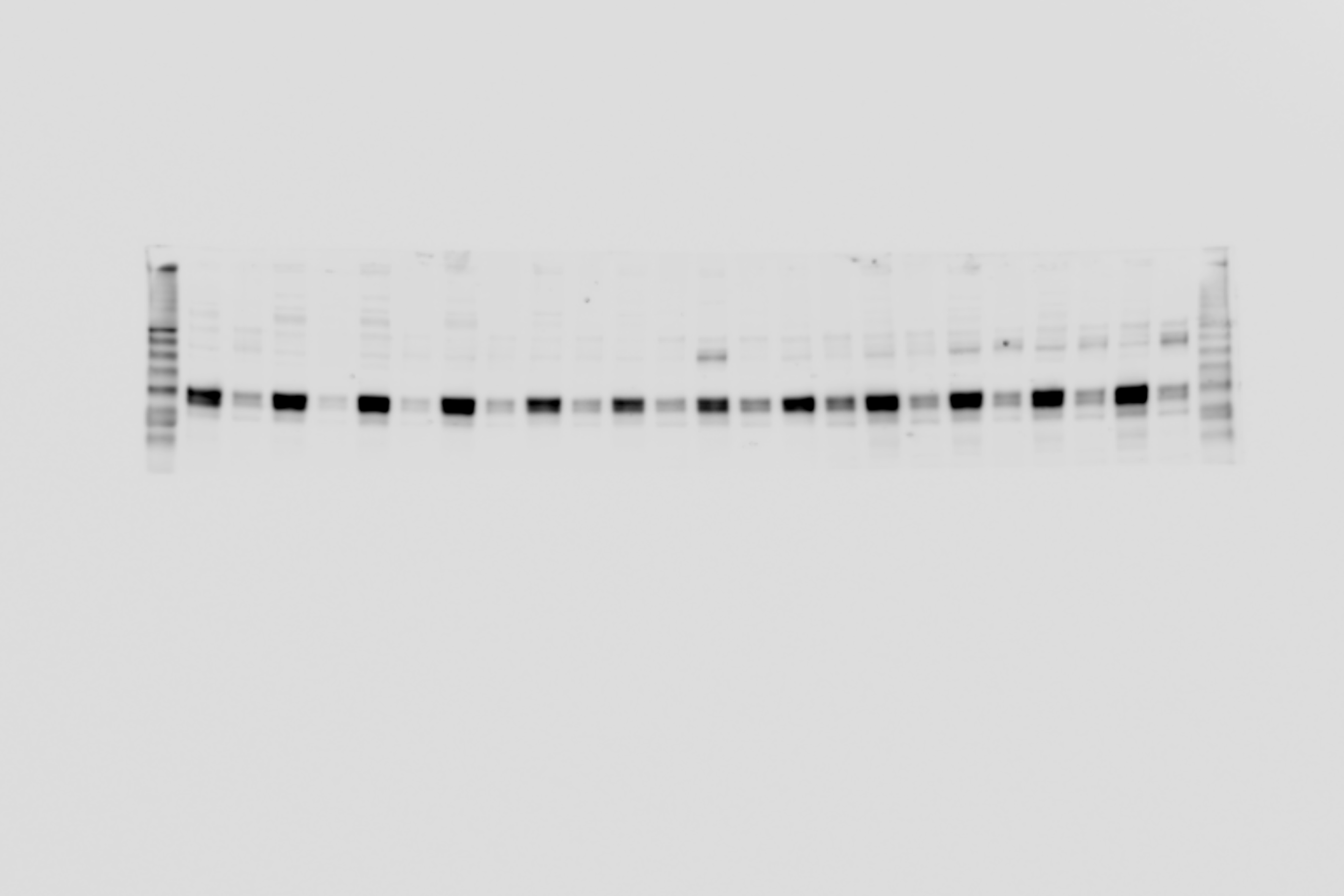

Supplement: Figure 6—source data 3. [file elife-85921-fig6-data3.zip › Fig6_SourceData3/Fig6G_CTX_Sp1_sourceblot.tif]

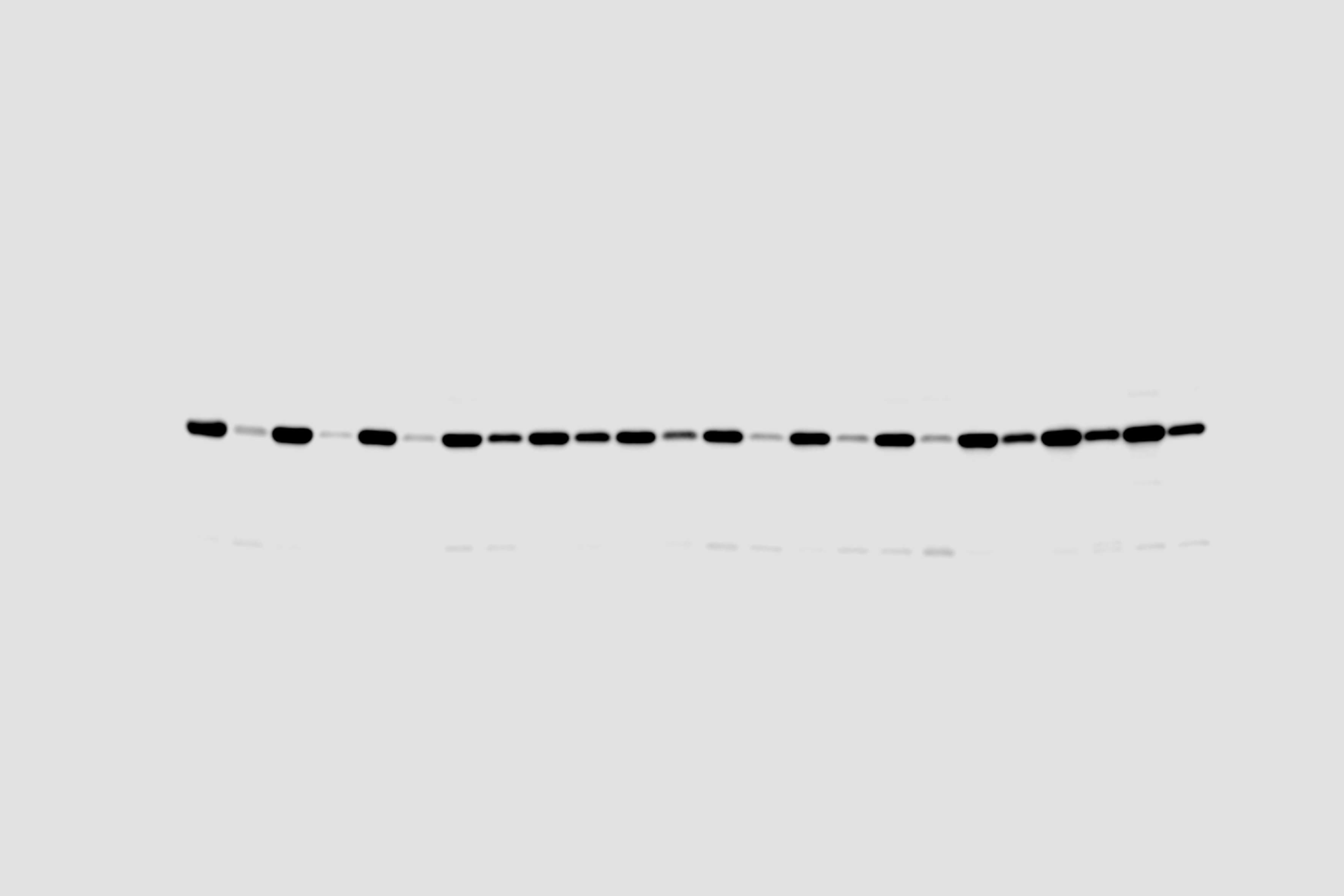

Supplement: Figure 6—source data 3. [file elife-85921-fig6-data3.zip › Fig6_SourceData3/Fig6G_CTX_TDP43_sourceblot.tif]

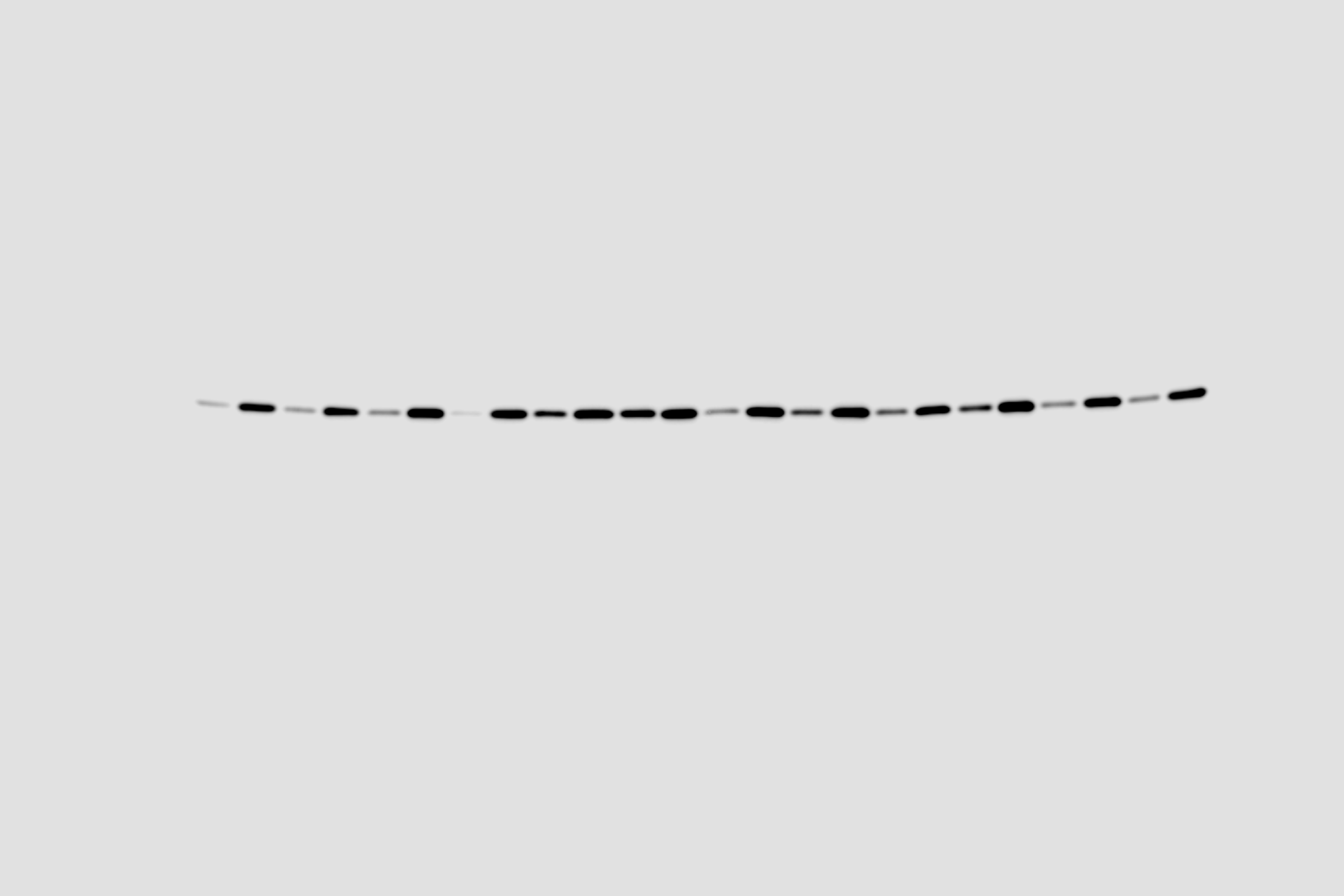

Supplement: Figure 6—source data 3. [file elife-85921-fig6-data3.zip › Fig6_SourceData3/Fig6J_Hipp_GAPDH_sourceblot.tif]

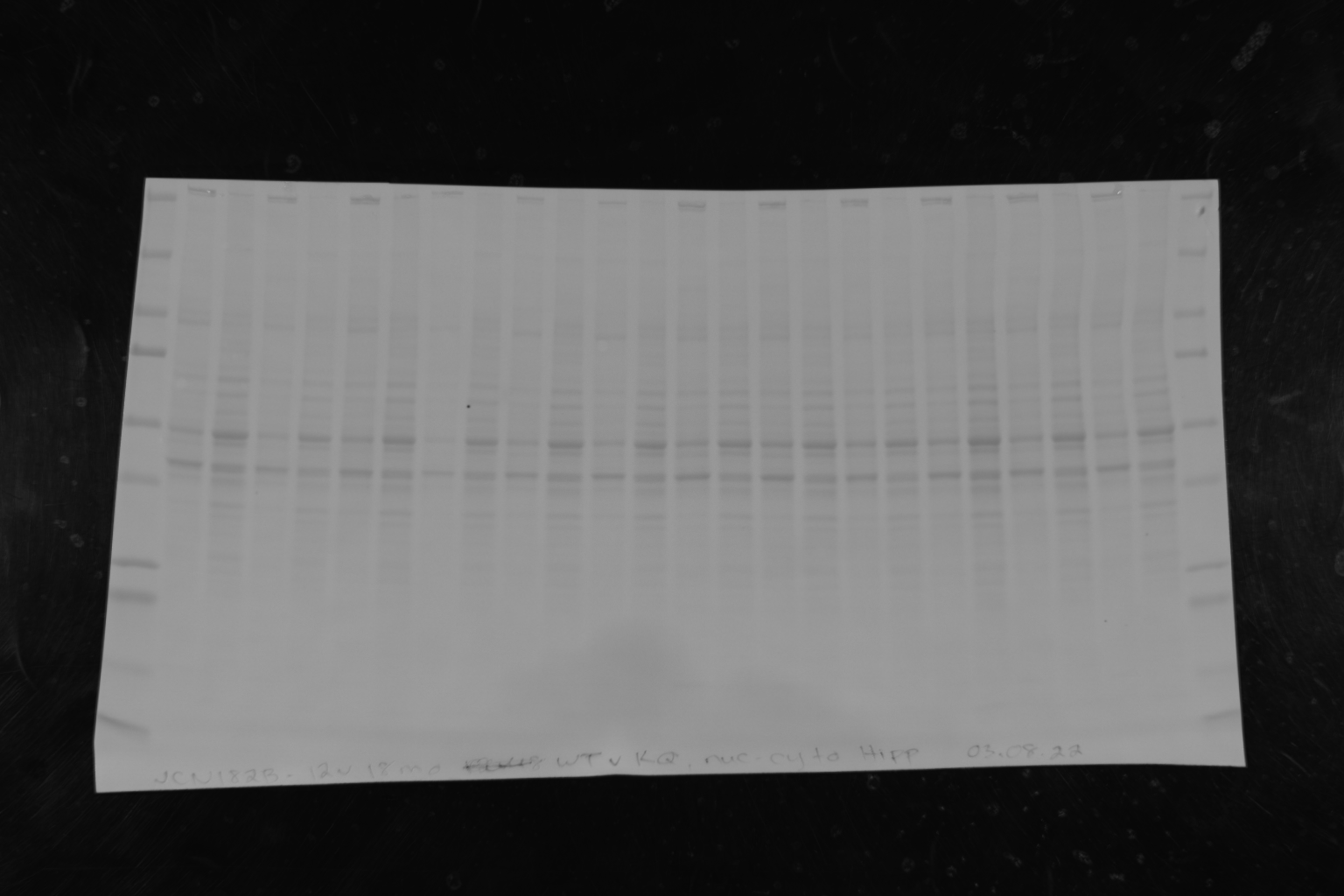

Supplement: Figure 6—source data 3. [file elife-85921-fig6-data3.zip › Fig6_SourceData3/Fig6J_Hipp_Ponceau-TTP_sourceblot.tif]

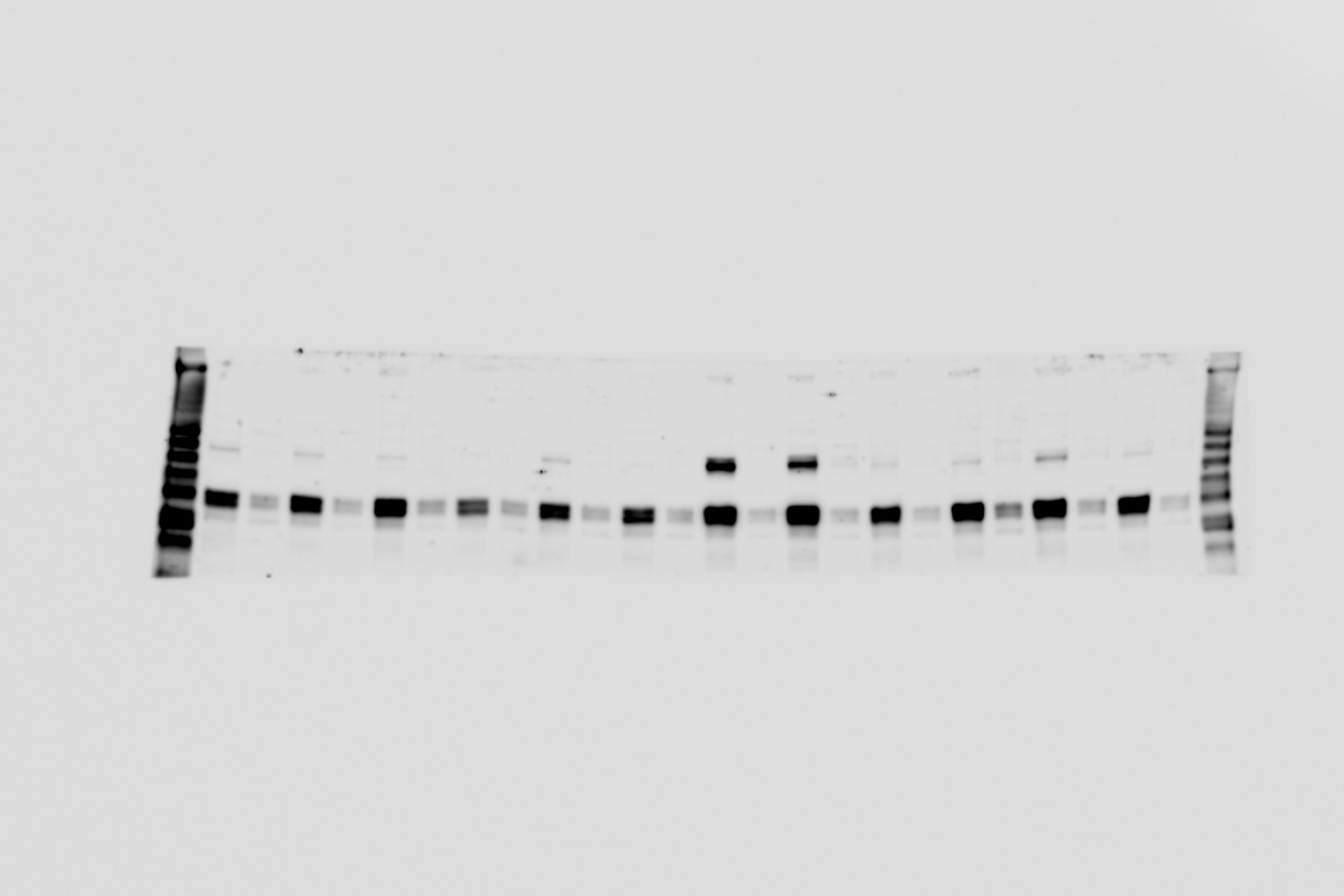

Supplement: Figure 6—source data 3. [file elife-85921-fig6-data3.zip › Fig6_SourceData3/Fig6J_Hipp_Sp1_sourceblot.tif]

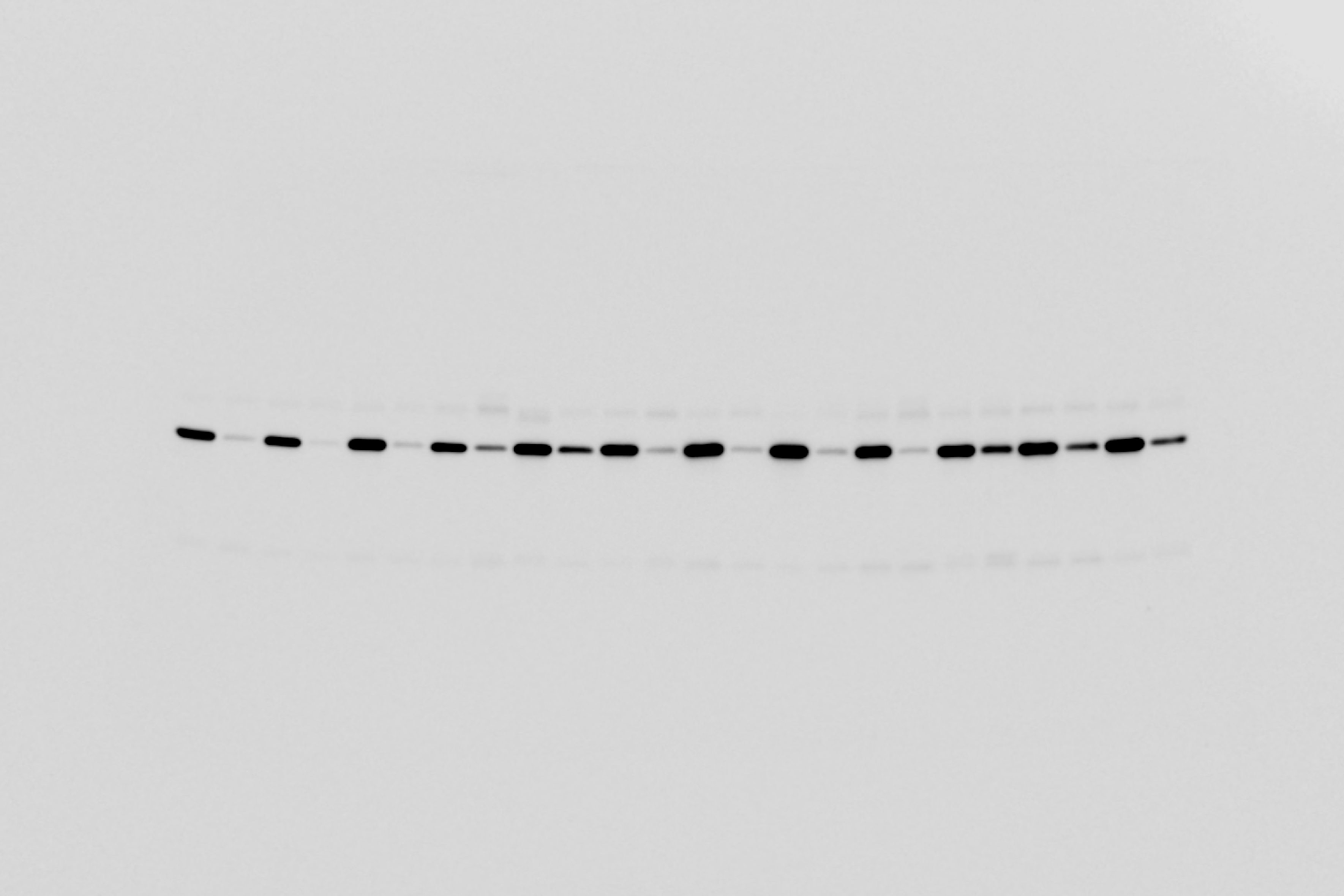

Supplement: Figure 6—source data 3. [file elife-85921-fig6-data3.zip › Fig6_SourceData3/Fig6J_Hipp_TDP43_sourceblot.tif]

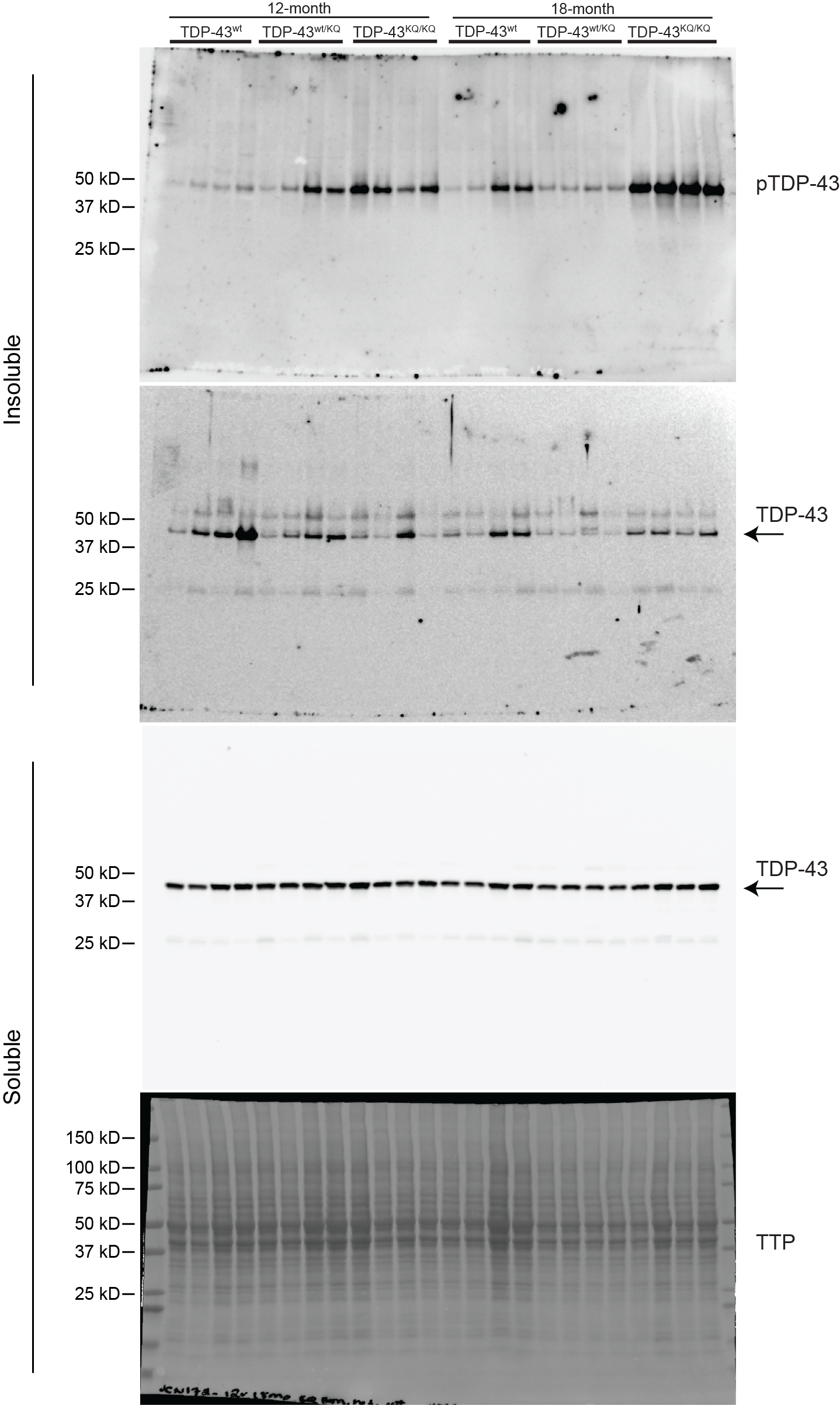

Supplement: Figure 6—figure supplement 1—source data 2. [file elife-85921-fig6-figsupp1-data2.zip › Fig6_SuppFig1_SourceData2/Fig6_SuppFig1_SourceData2_A.png]

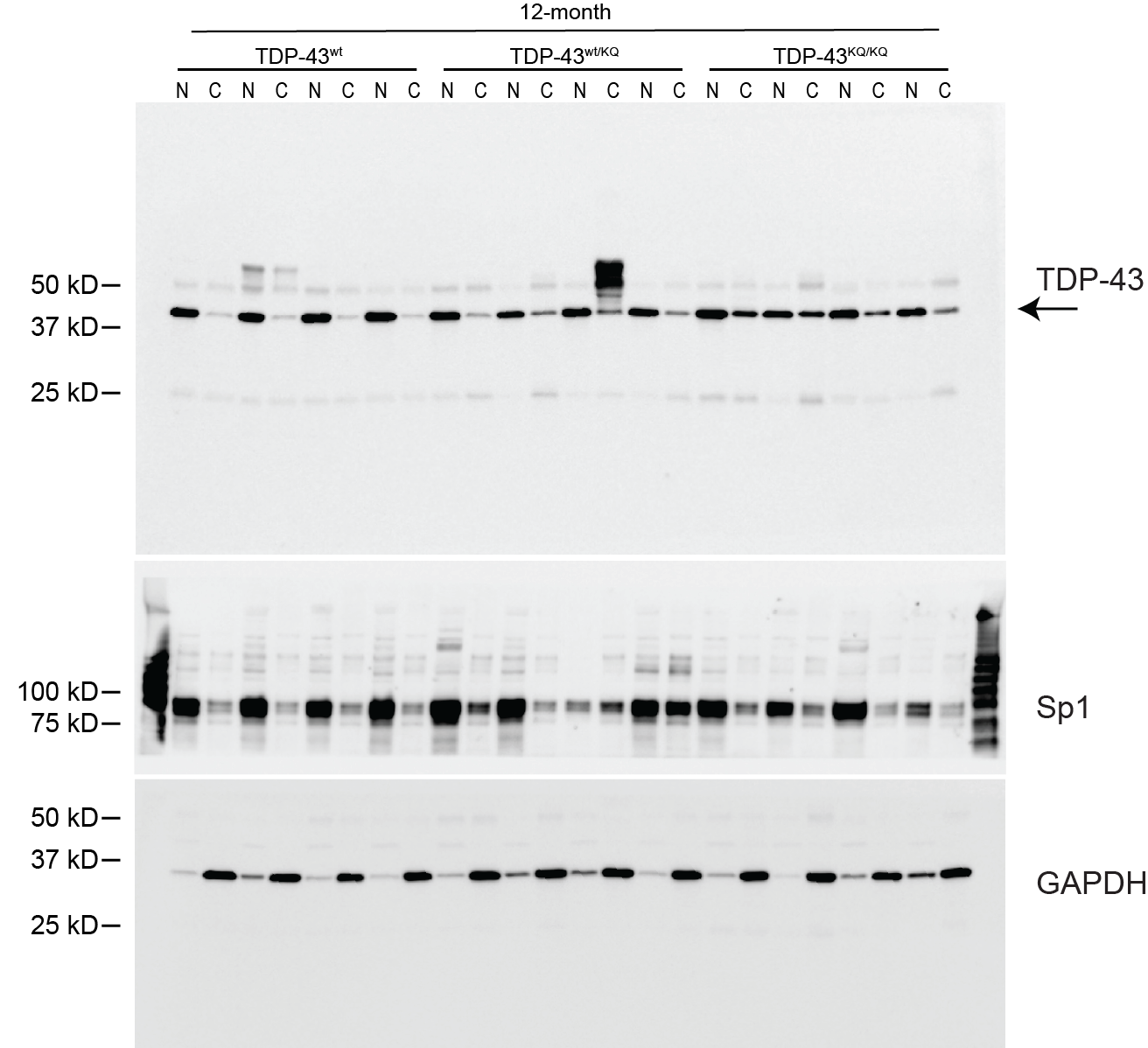

Supplement: Figure 6—figure supplement 1—source data 2. [file elife-85921-fig6-figsupp1-data2.zip › Fig6_SuppFig1_SourceData2/Fig6_SuppFig1_SourceData2_D.png]

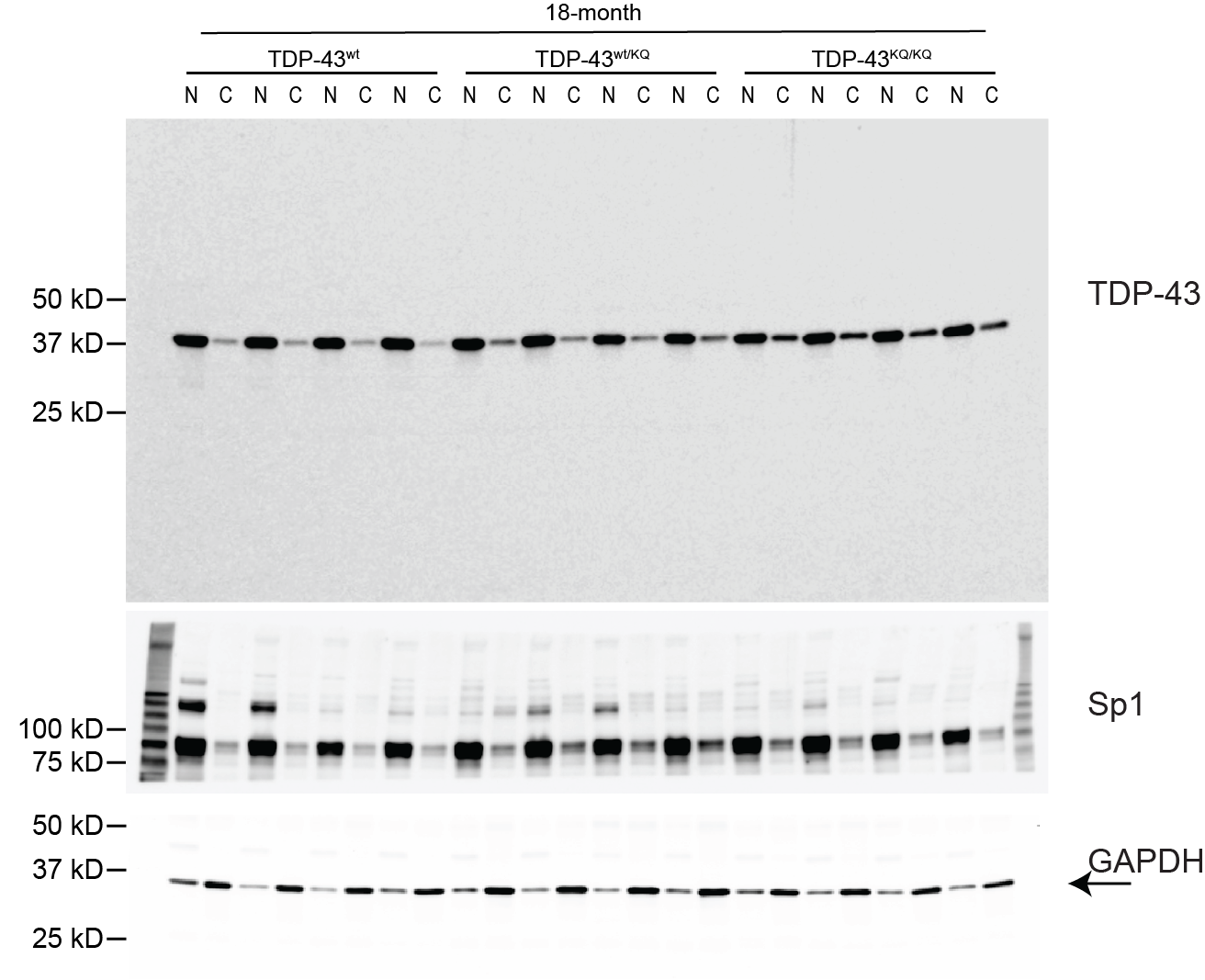

Supplement: Figure 6—figure supplement 1—source data 2. [file elife-85921-fig6-figsupp1-data2.zip › Fig6_SuppFig1_SourceData2/Fig6_SuppFig1_SourceData2_G.png]

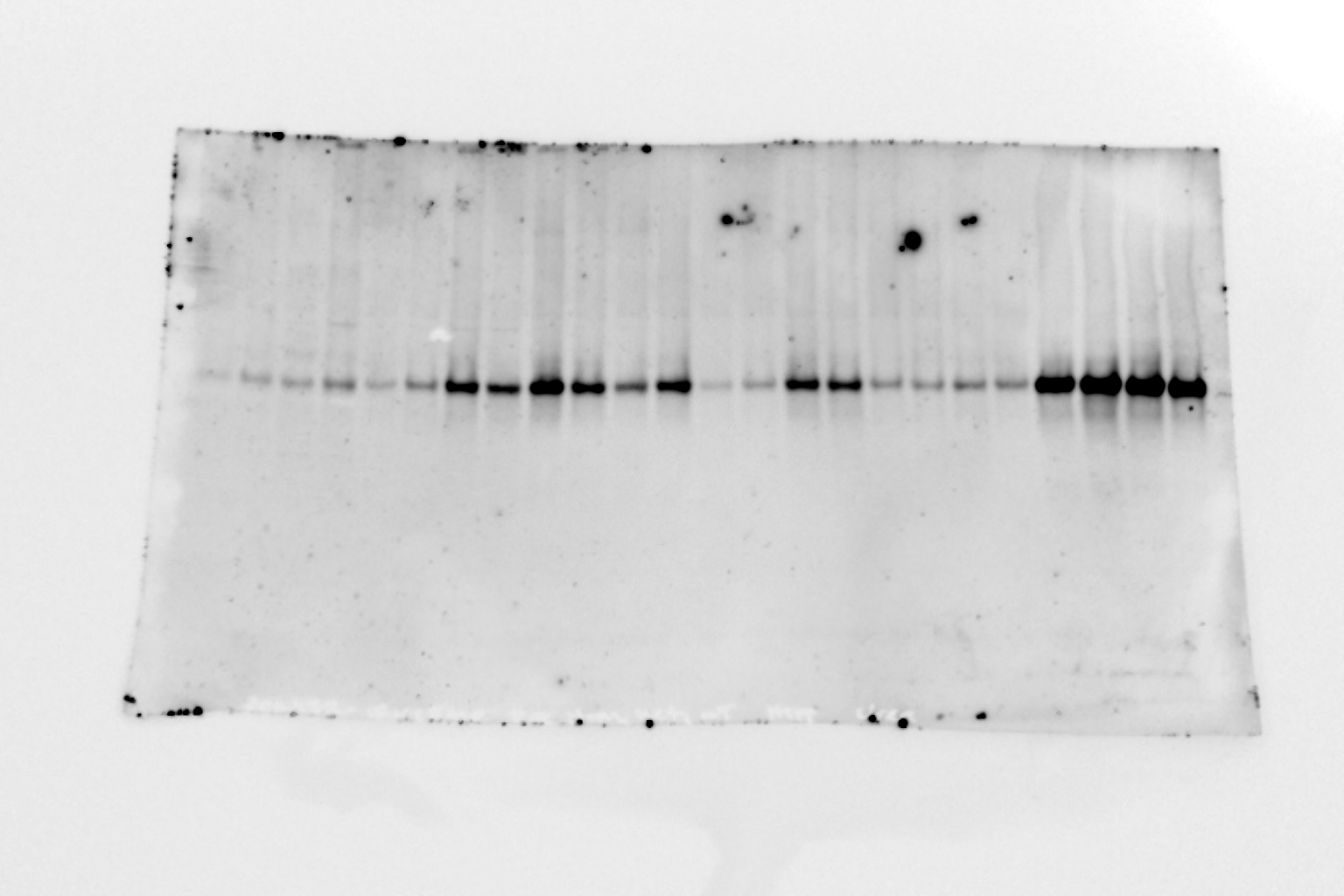

Supplement: Figure 6—figure supplement 1—source data 3. [file elife-85921-fig6-figsupp1-data3.zip › Fig6_SuppFig1_SourceData5/Fig6_SuppFig1A_Hipp_Insolp409.410TDP43_sourceblot.tif]

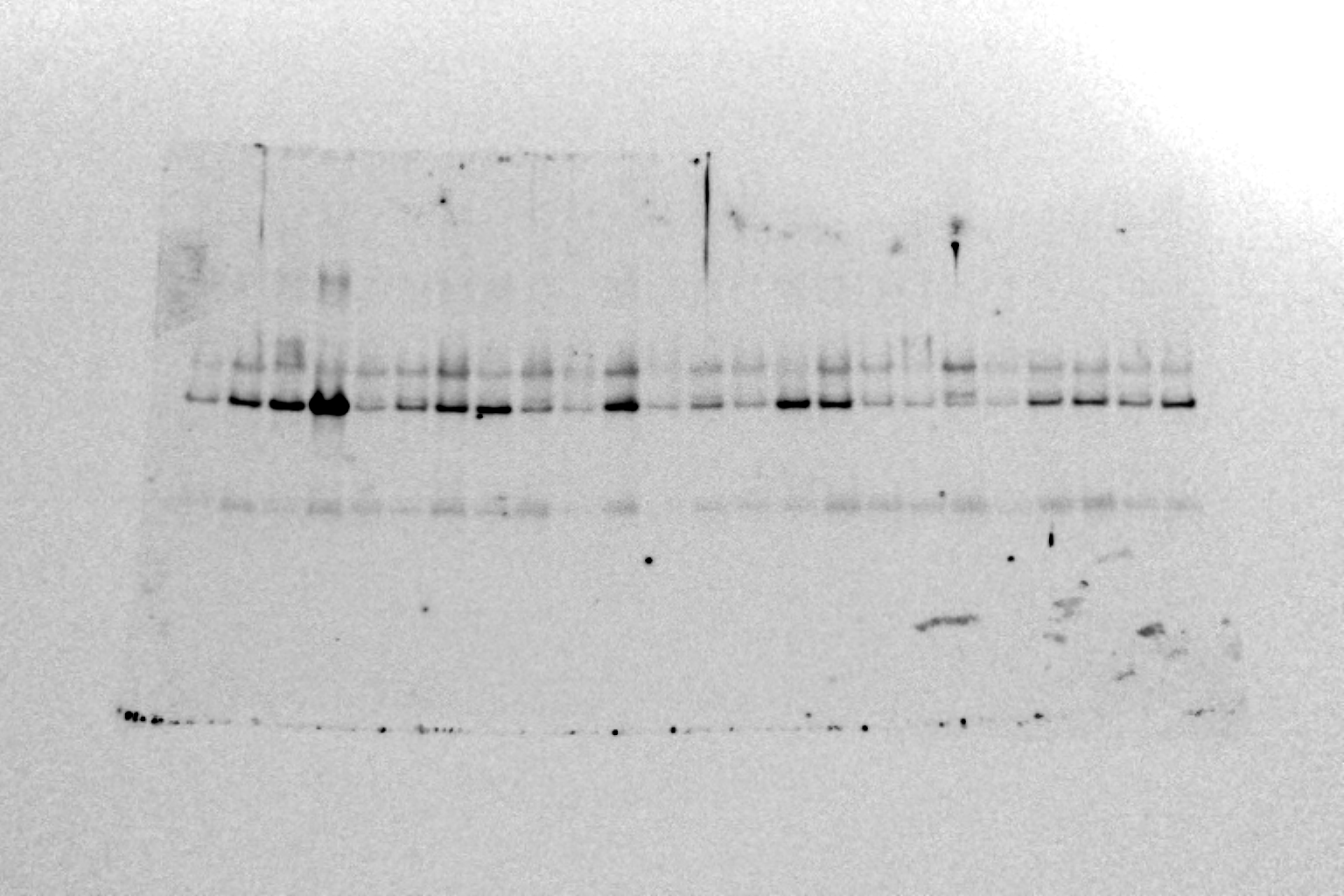

Supplement: Figure 6—figure supplement 1—source data 3. [file elife-85921-fig6-figsupp1-data3.zip › Fig6_SuppFig1_SourceData5/Fig6_SuppFig1A_Hipp_InsolTDP43_sourceblot.tif]

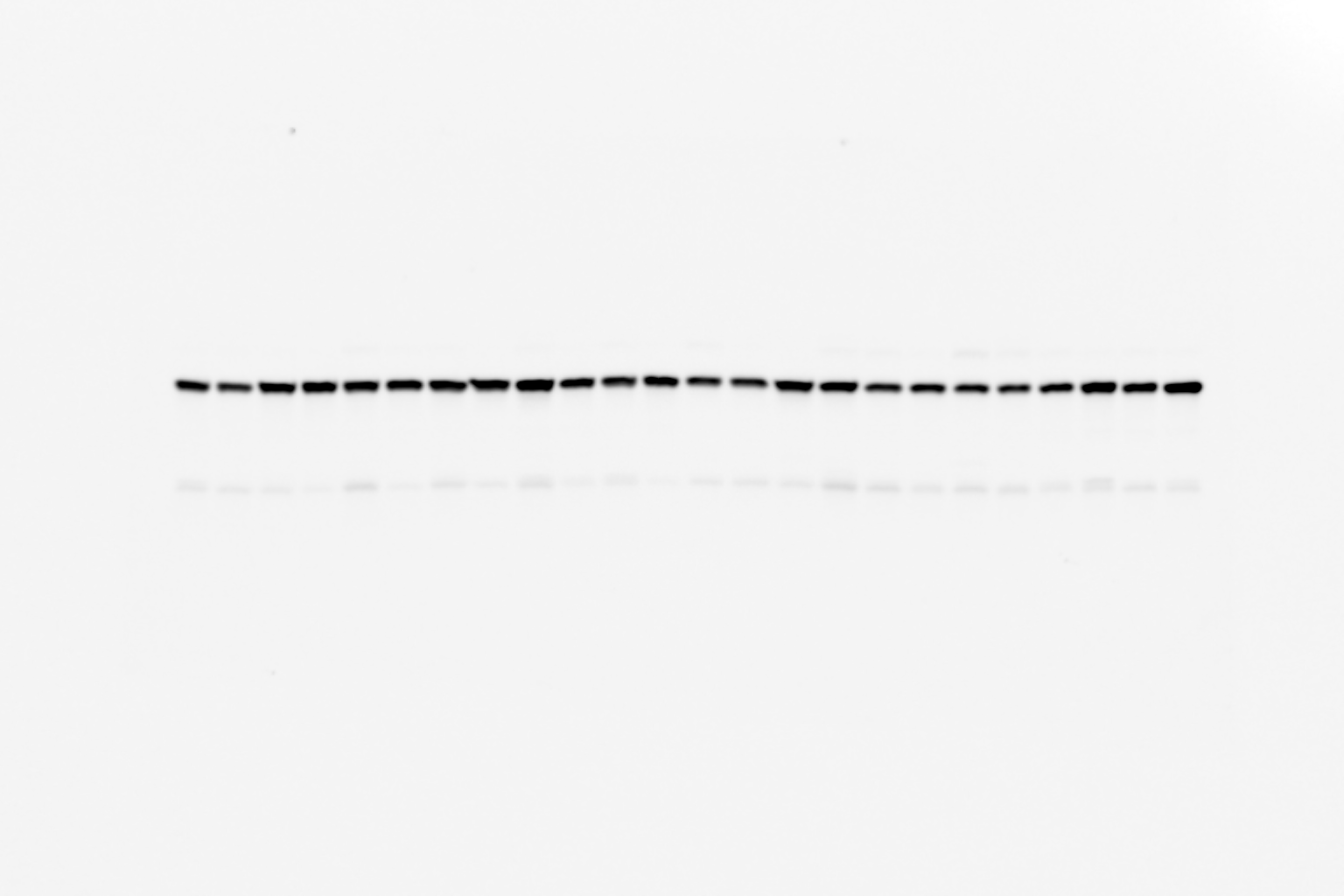

Supplement: Figure 6—figure supplement 1—source data 3. [file elife-85921-fig6-figsupp1-data3.zip › Fig6_SuppFig1_SourceData5/Fig6_SuppFig1A_Hipp_TDP43_sourceblot.tif]

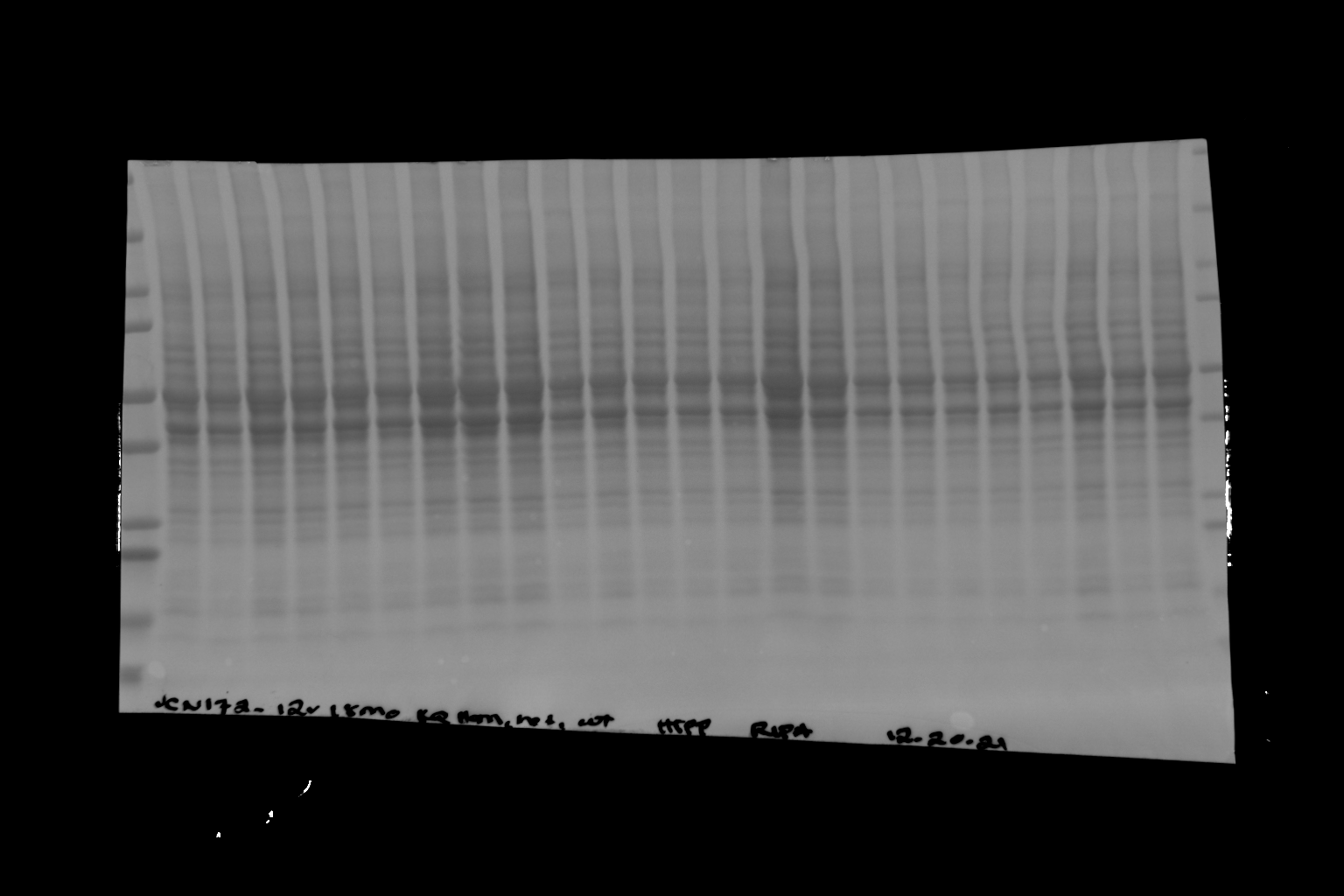

Supplement: Figure 6—figure supplement 1—source data 3. [file elife-85921-fig6-figsupp1-data3.zip › Fig6_SuppFig1_SourceData5/Fig6_SuppFig1A_Hipp_TTP_sourceblot.tif]

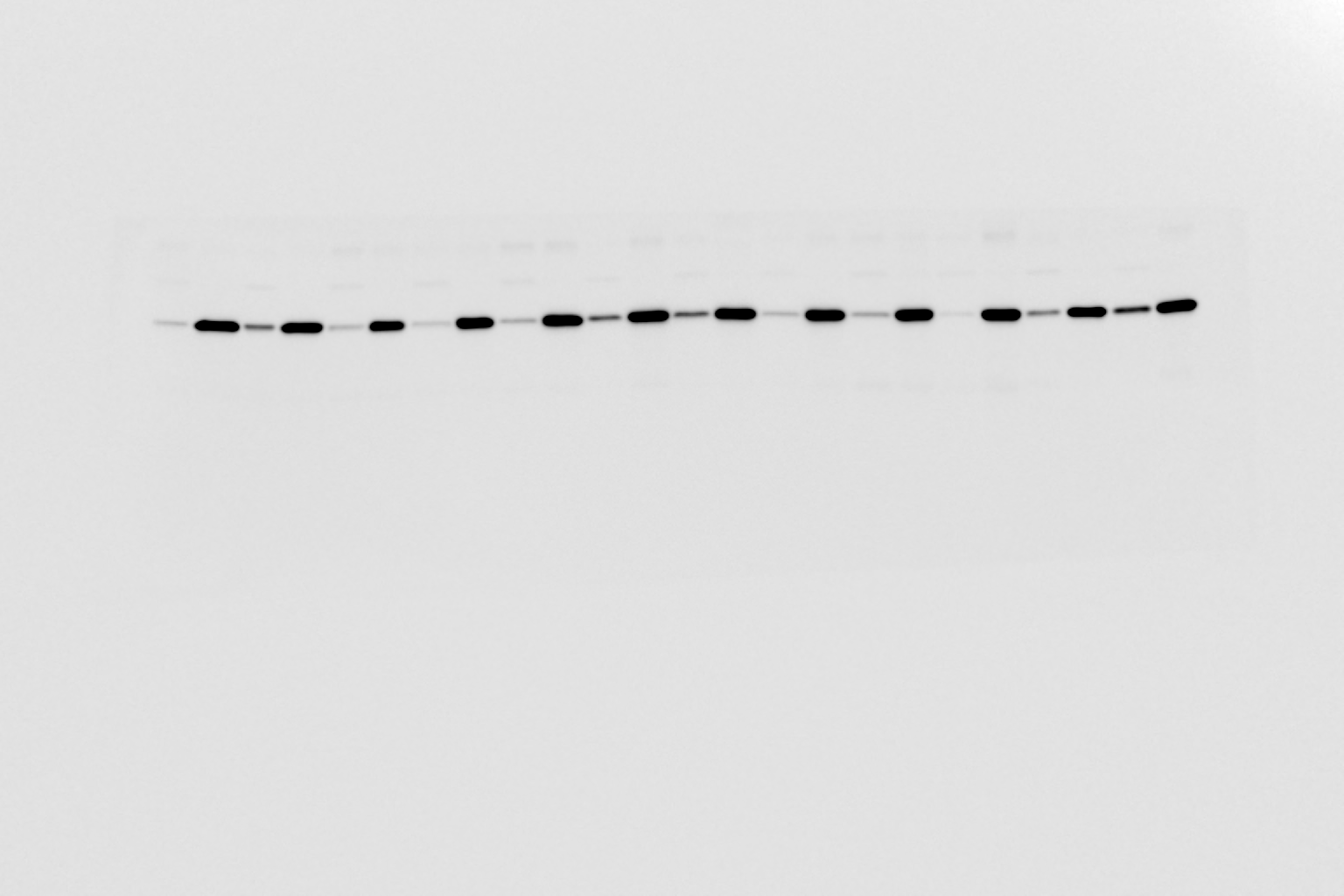

Supplement: Figure 6—figure supplement 1—source data 3. [file elife-85921-fig6-figsupp1-data3.zip › Fig6_SuppFig1_SourceData5/Fig6_SuppFig1D_Hipp_GAPDH_sourceblot.tif]

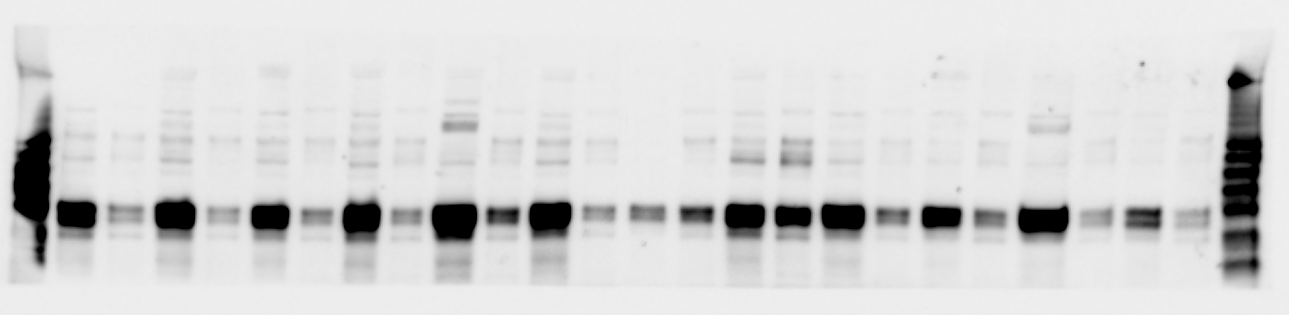

Supplement: Figure 6—figure supplement 1—source data 3. [file elife-85921-fig6-figsupp1-data3.zip › Fig6_SuppFig1_SourceData5/Fig6_SuppFig1D_Hipp_Sp1_sourceblot.tif]

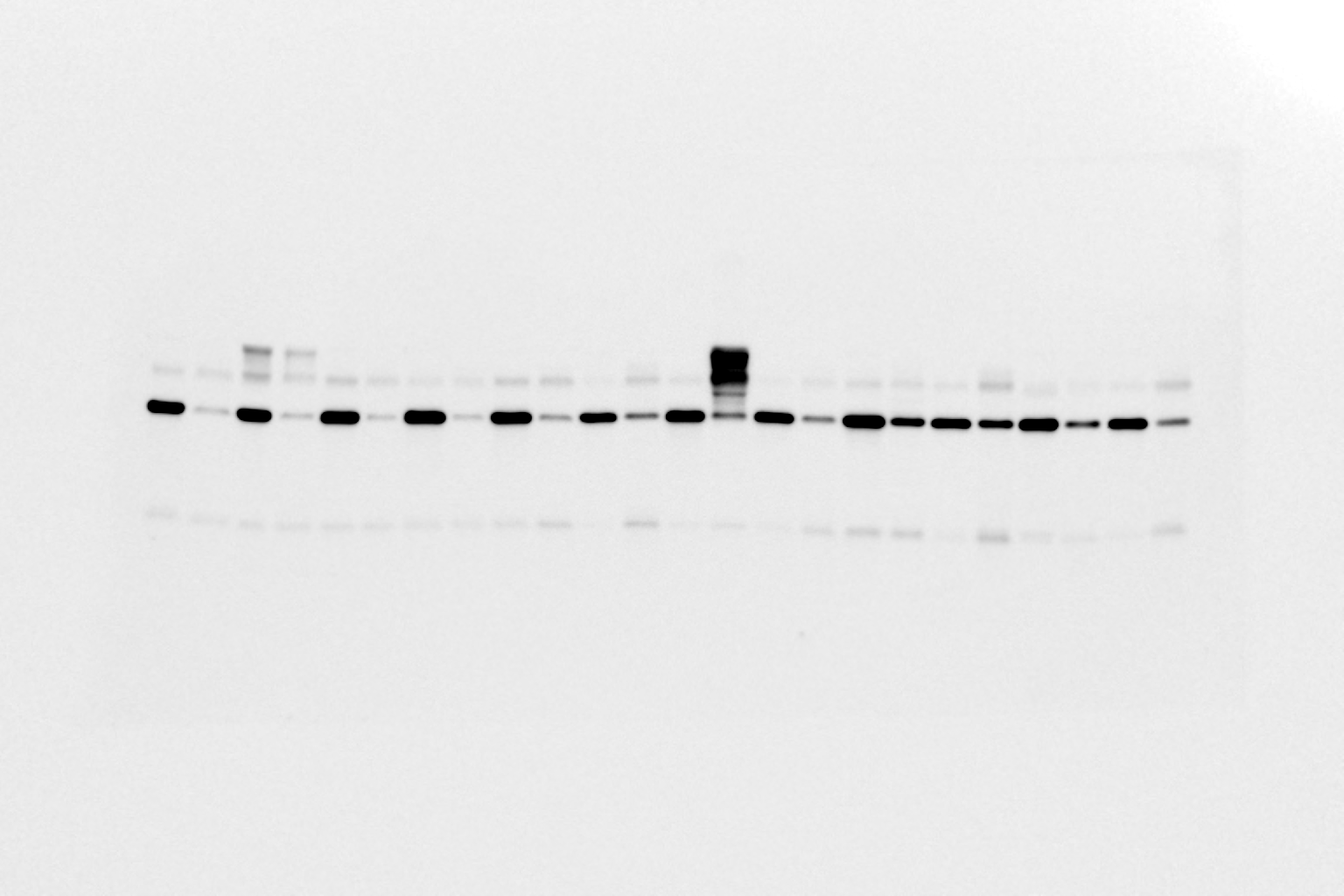

Supplement: Figure 6—figure supplement 1—source data 3. [file elife-85921-fig6-figsupp1-data3.zip › Fig6_SuppFig1_SourceData5/Fig6_SuppFig1D_Hipp_TDP43_sourceblot.tif]

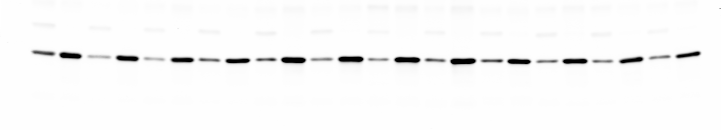

Supplement: Figure 6—figure supplement 1—source data 3. [file elife-85921-fig6-figsupp1-data3.zip › Fig6_SuppFig1_SourceData5/Fig6_SuppFig1G_Hipp_GAPDH_sourceblot.tif]

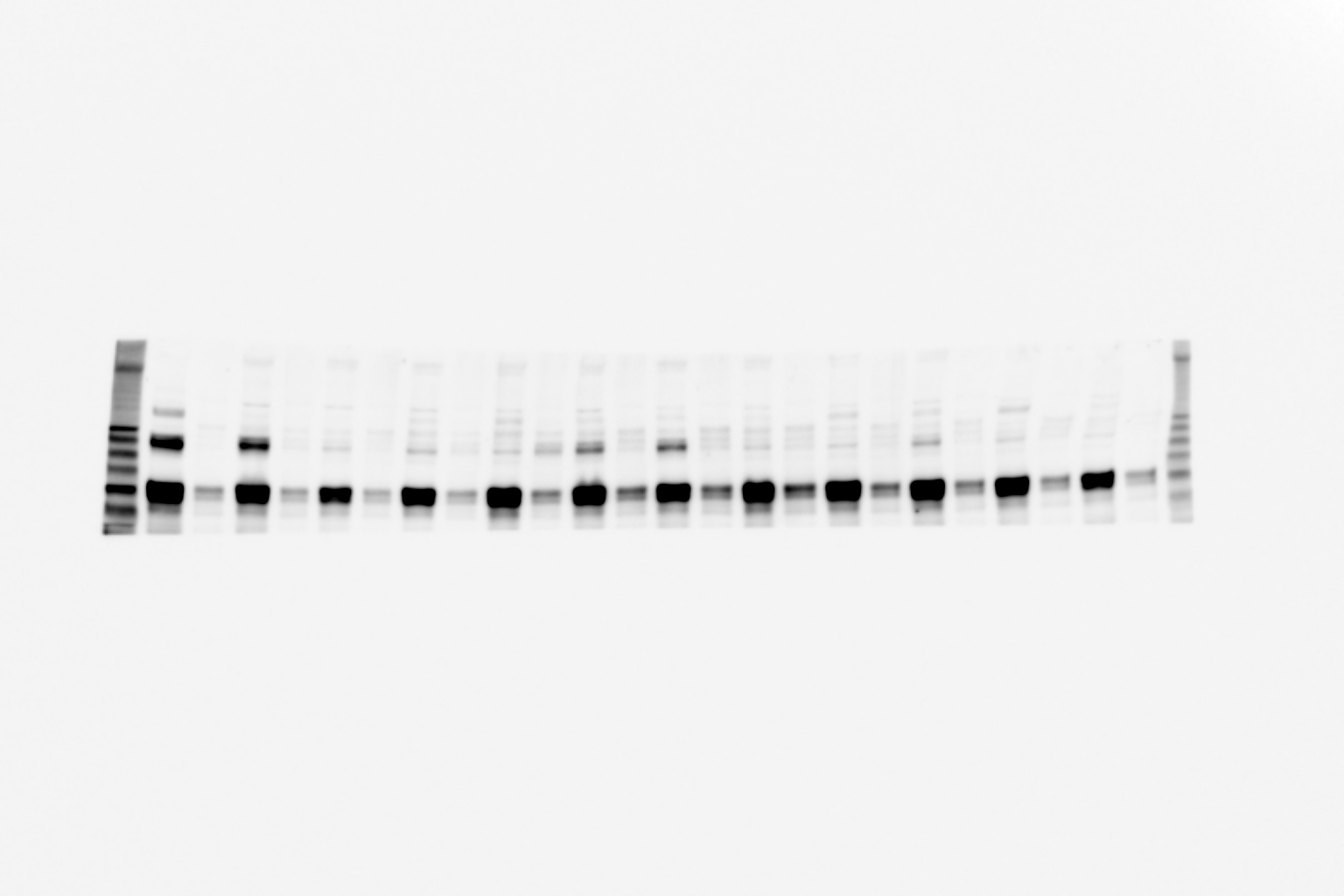

Supplement: Figure 6—figure supplement 1—source data 3. [file elife-85921-fig6-figsupp1-data3.zip › Fig6_SuppFig1_SourceData5/Fig6_SuppFig1G_Hipp_Sp1_sourceblot.tif]

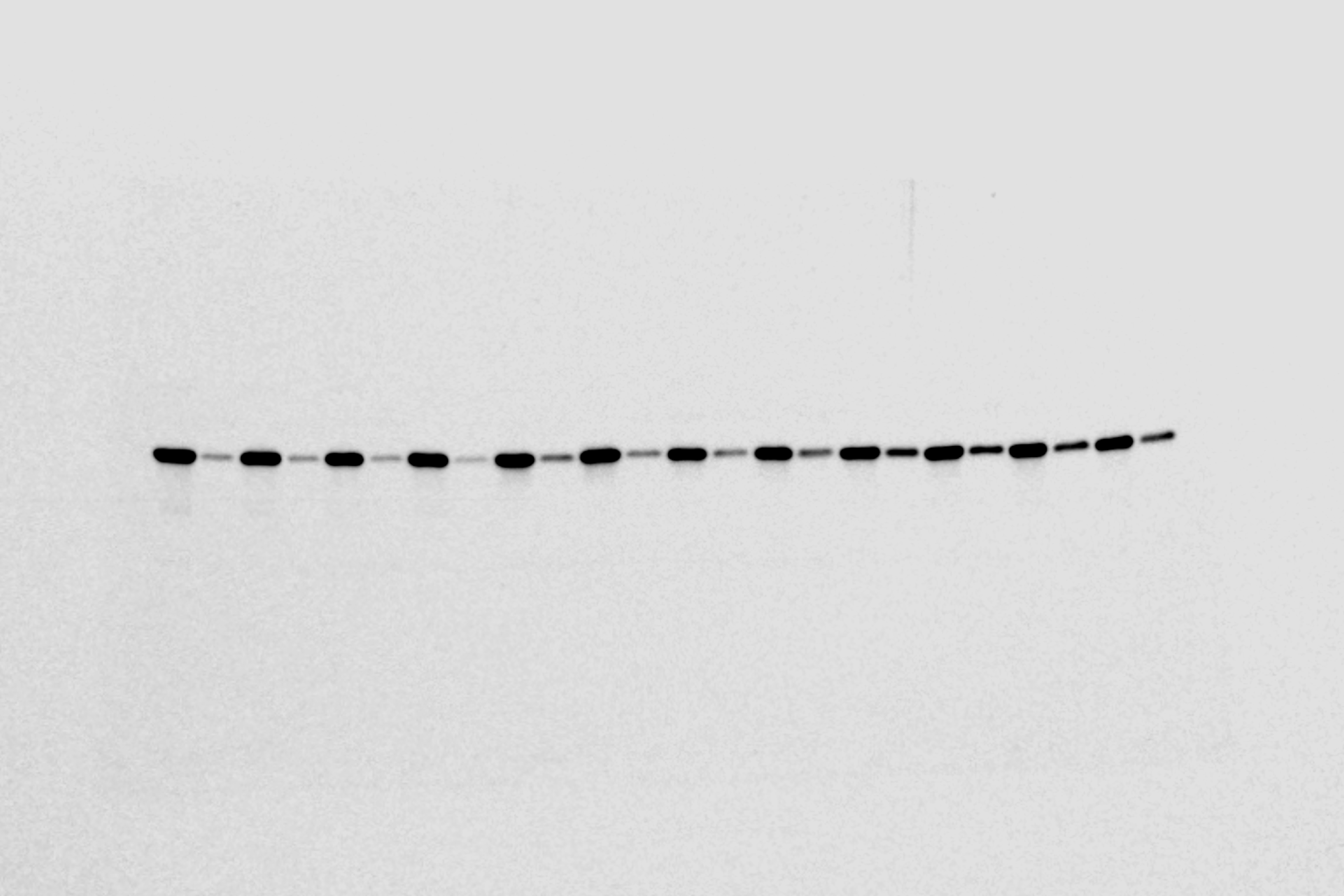

Supplement: Figure 6—figure supplement 1—source data 3. [file elife-85921-fig6-figsupp1-data3.zip › Fig6_SuppFig1_SourceData5/Fig6_SuppFig1G_Hipp_TDP43_sourceblot.tif]

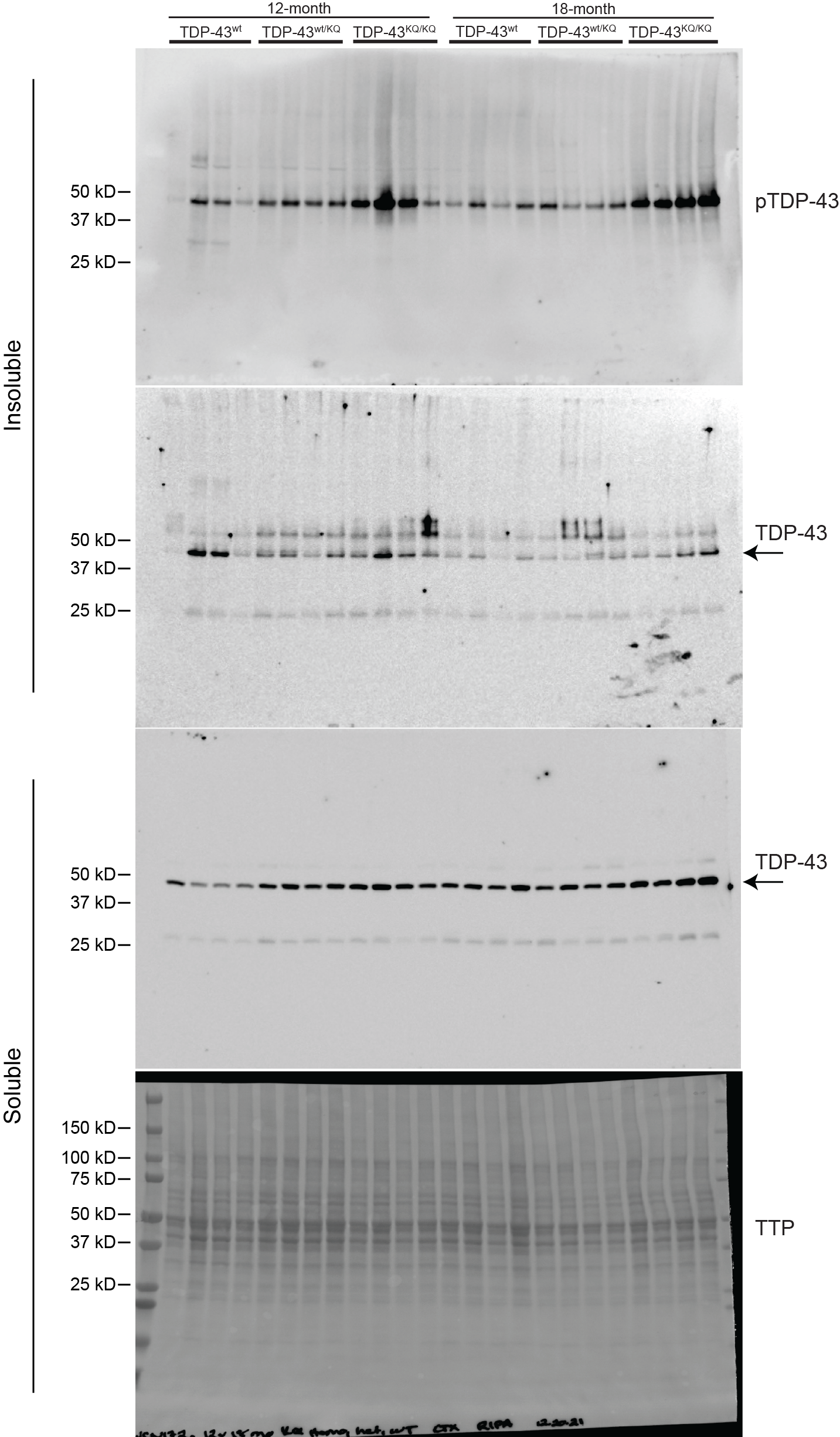

Supplement: Figure 6—figure supplement 2—source data 2. [file elife-85921-fig6-figsupp2-data2.zip › Fig6_SuppFig2_SourceData2/Fig6_SuppFig2_SourceData2_A.png]

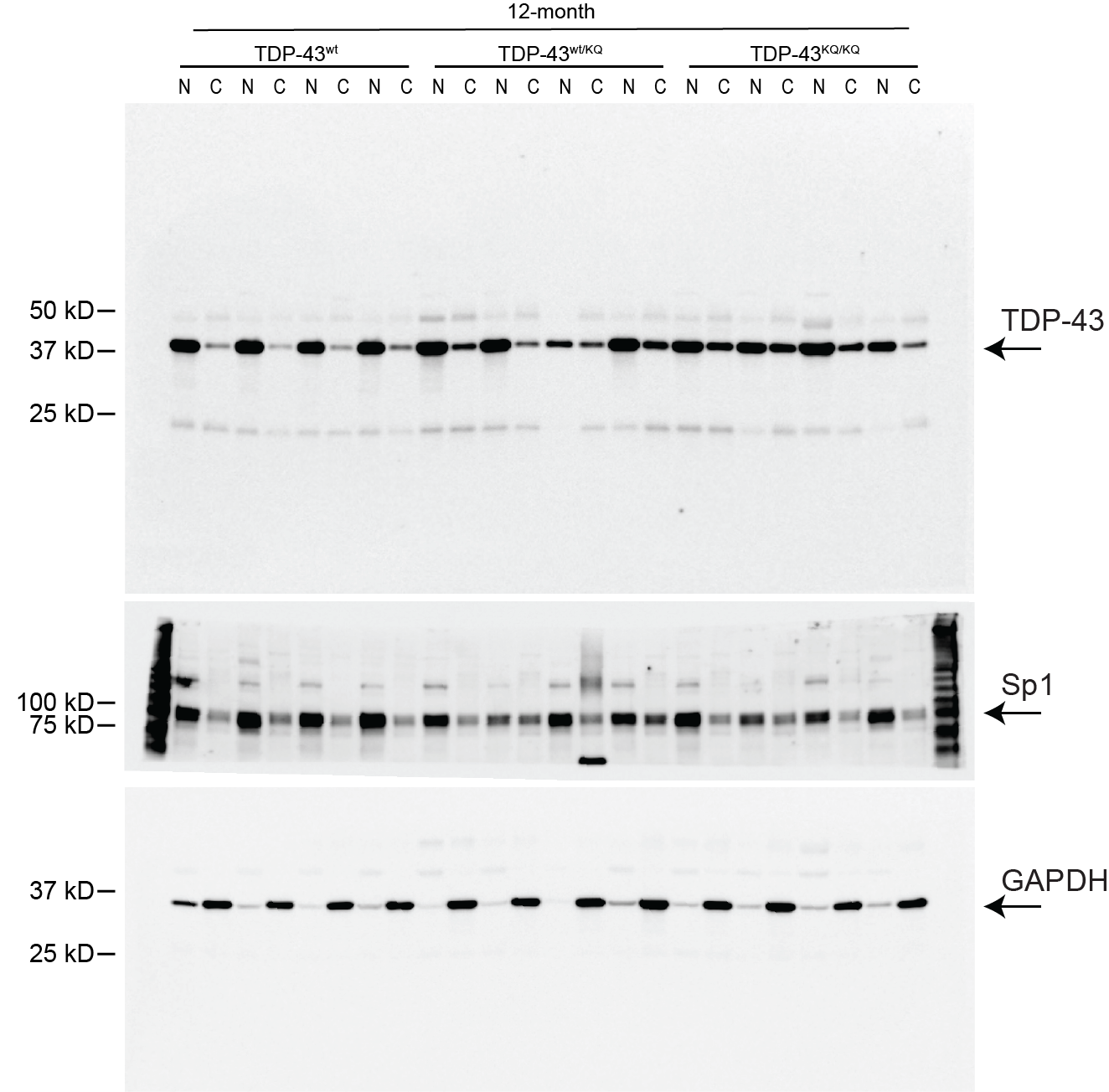

Supplement: Figure 6—figure supplement 2—source data 2. [file elife-85921-fig6-figsupp2-data2.zip › Fig6_SuppFig2_SourceData2/Fig6_SuppFig2_SourceData2_D.png]

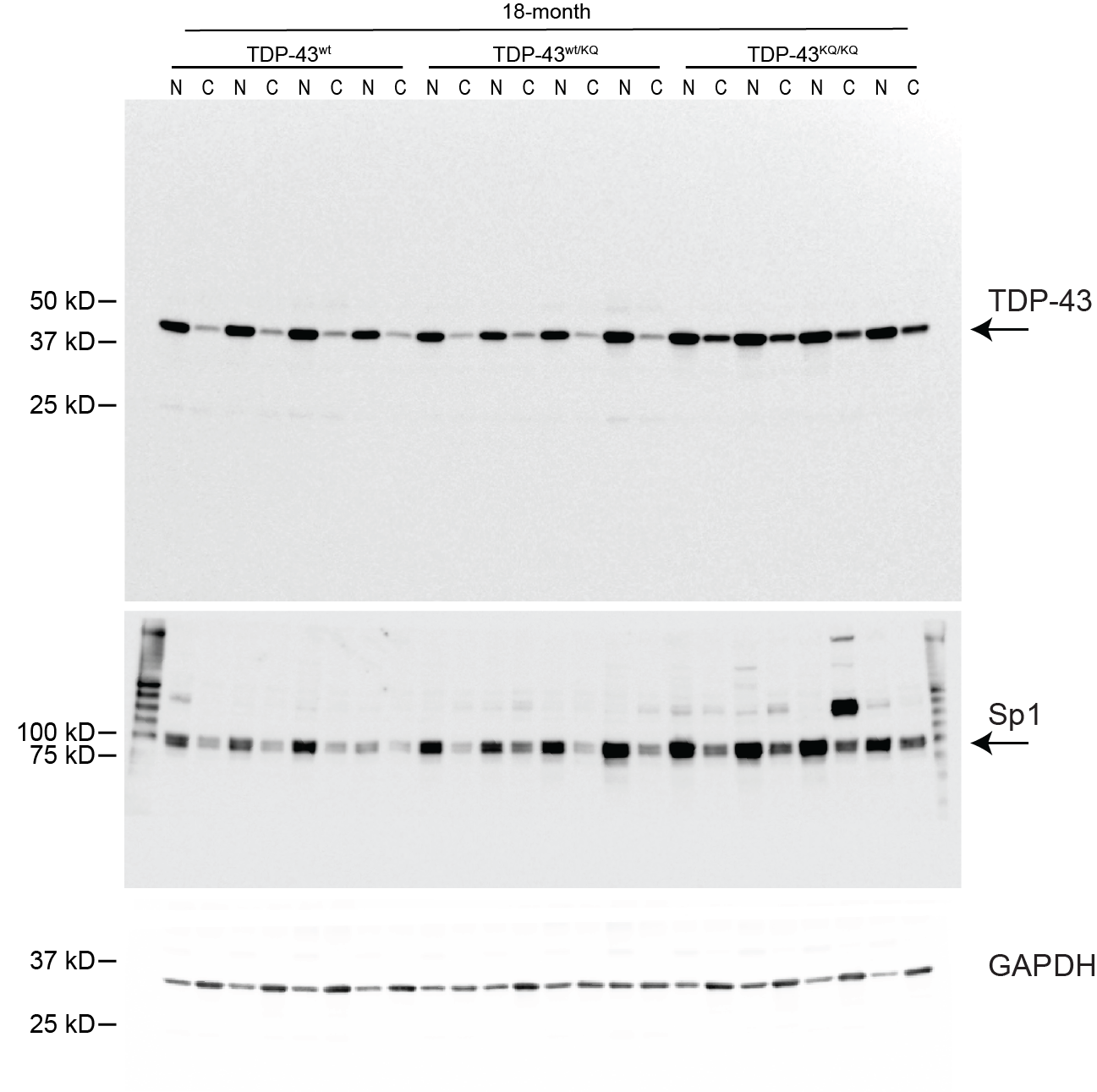

Supplement: Figure 6—figure supplement 2—source data 2. [file elife-85921-fig6-figsupp2-data2.zip › Fig6_SuppFig2_SourceData2/Fig6_SuppFig2_SourceData2_G.png]

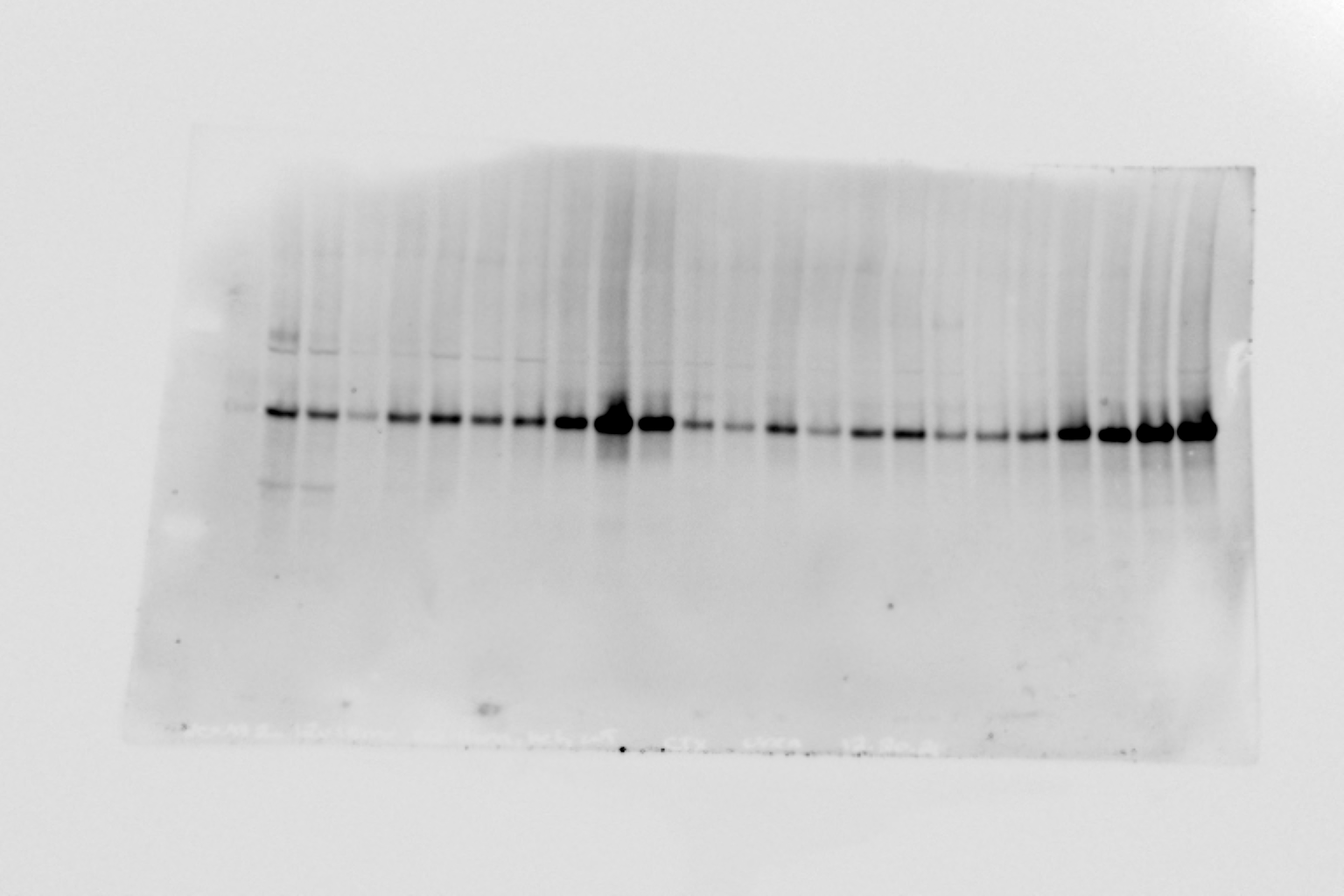

Supplement: Figure 6—figure supplement 2—source data 3. [file elife-85921-fig6-figsupp2-data3.zip › Fig6_SuppFig2_SourceData5/Fig6_SuppFig2A_CTX_insolp409.410TDP_sourceblot.tif]

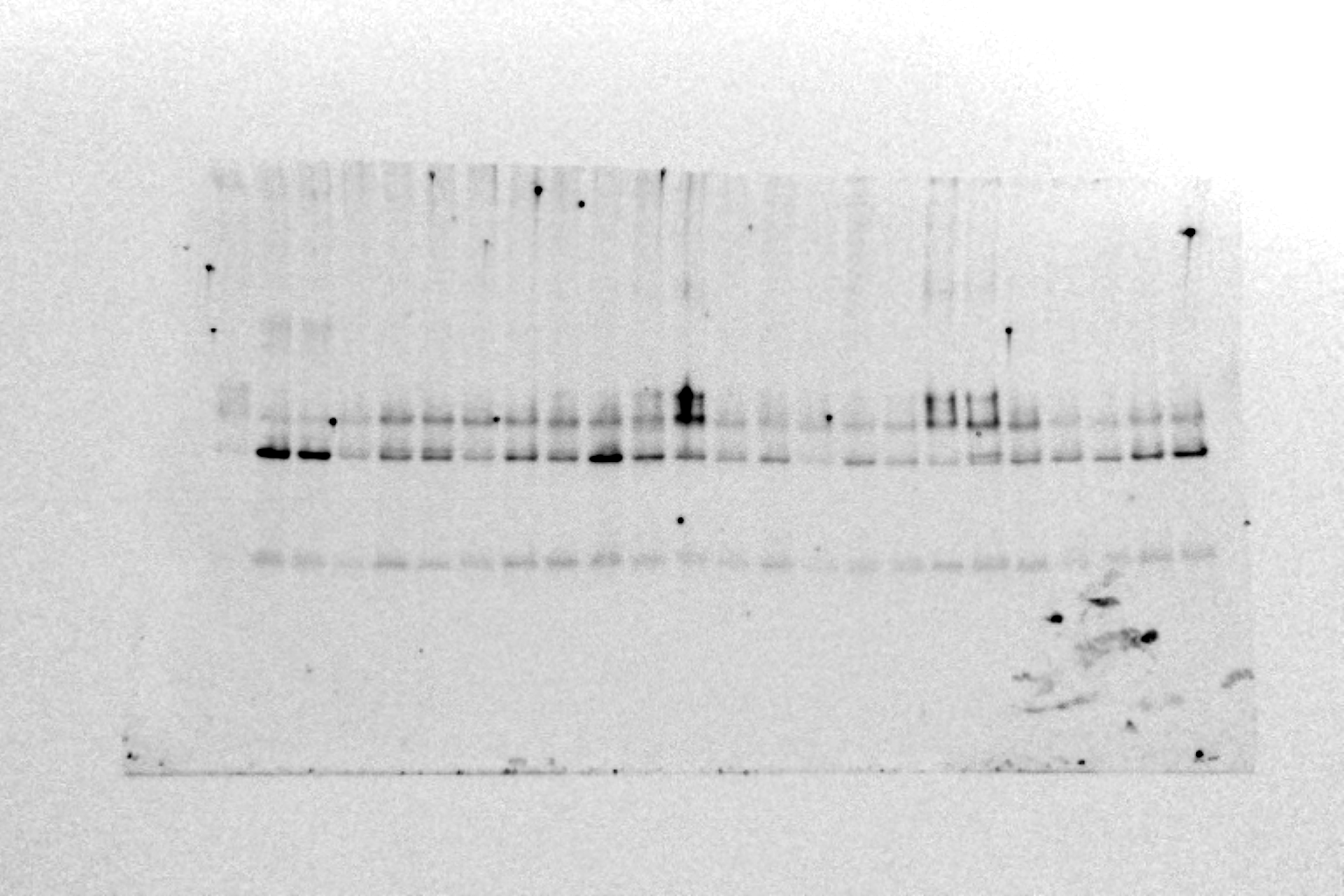

Supplement: Figure 6—figure supplement 2—source data 3. [file elife-85921-fig6-figsupp2-data3.zip › Fig6_SuppFig2_SourceData5/Fig6_SuppFig2A_CTX_insolTDP43_sourceblot.tif]

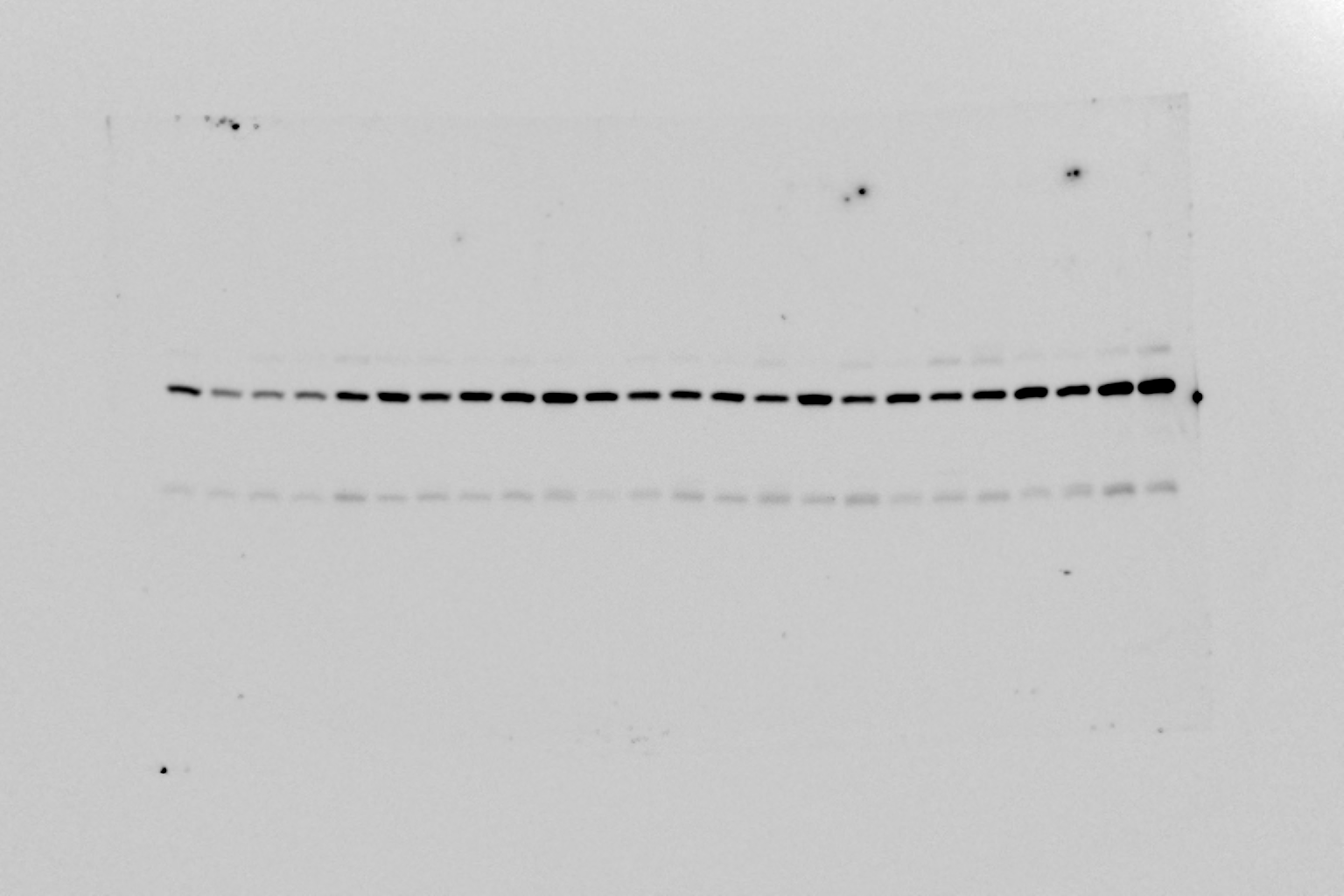

Supplement: Figure 6—figure supplement 2—source data 3. [file elife-85921-fig6-figsupp2-data3.zip › Fig6_SuppFig2_SourceData5/Fig6_SuppFig2A_CTX_TDP43_sourceblot.tif]

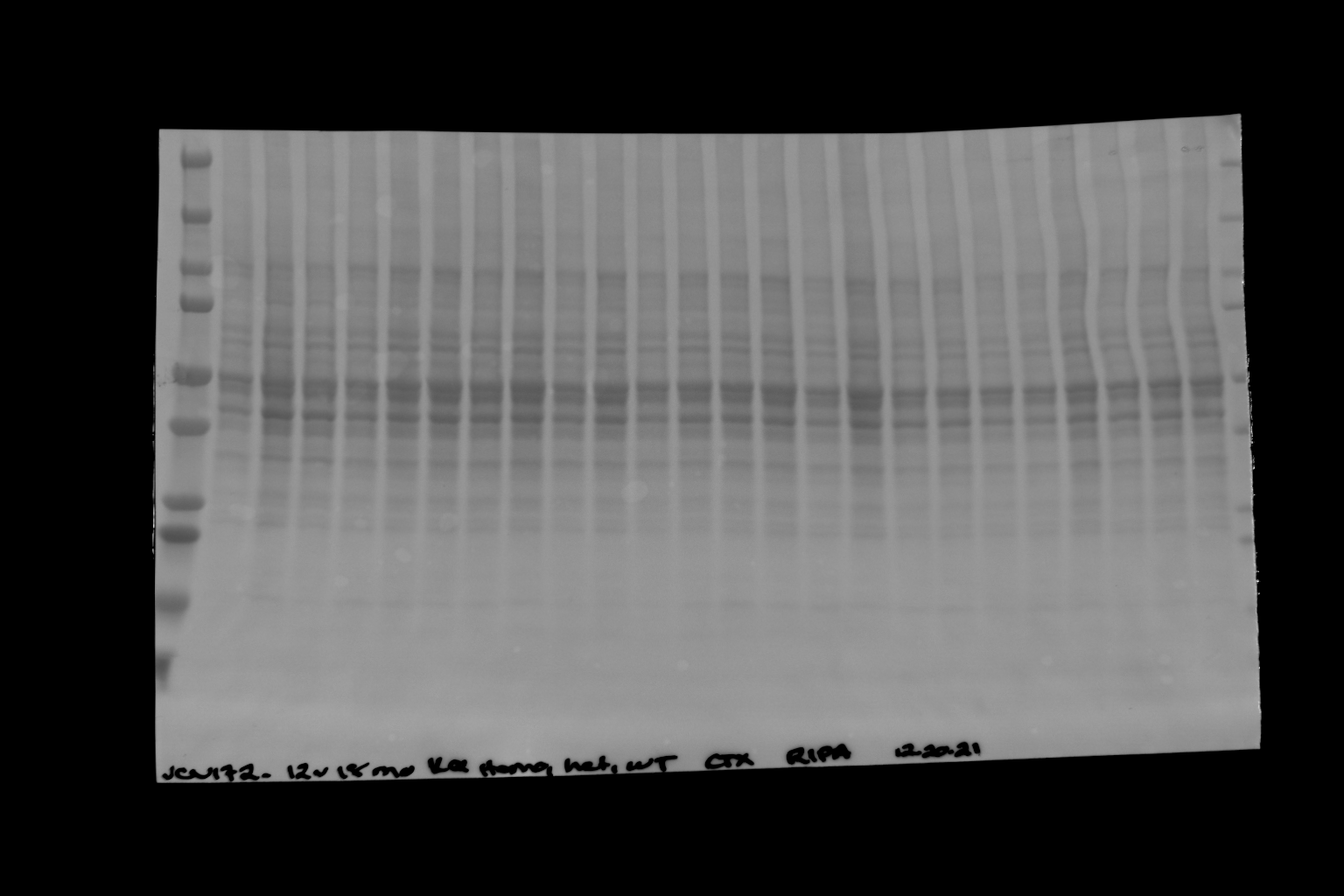

Supplement: Figure 6—figure supplement 2—source data 3. [file elife-85921-fig6-figsupp2-data3.zip › Fig6_SuppFig2_SourceData5/Fig6_SuppFig2A_CTX_TTP_sourceblot.tif]

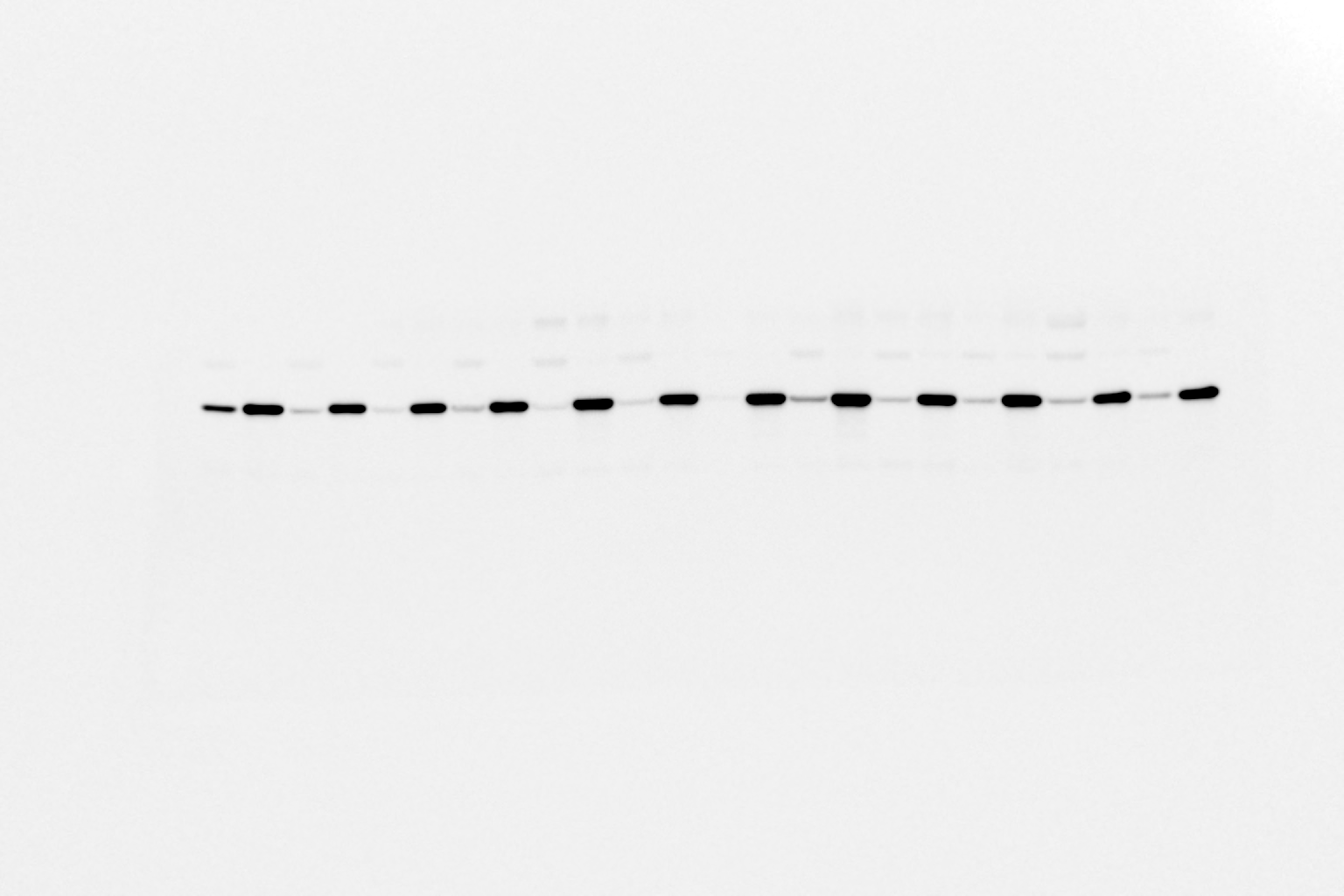

Supplement: Figure 6—figure supplement 2—source data 3. [file elife-85921-fig6-figsupp2-data3.zip › Fig6_SuppFig2_SourceData5/Fig6_SuppFig2D_CTX_GAPDH_sourceblot.tif]

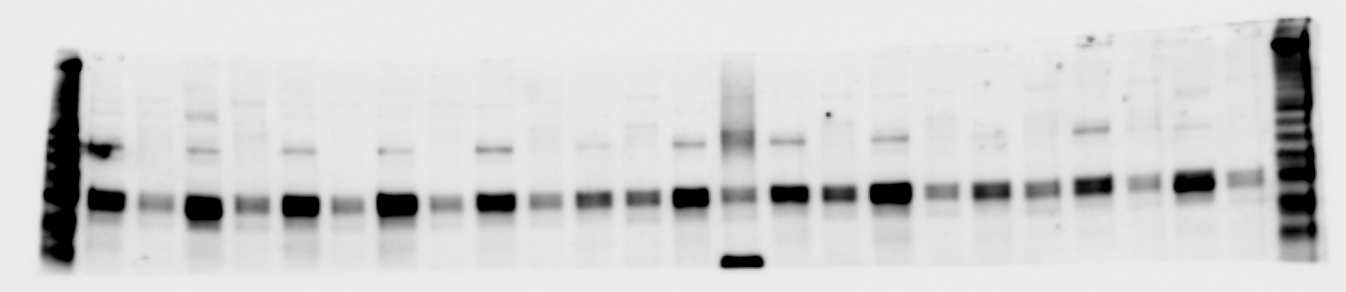

Supplement: Figure 6—figure supplement 2—source data 3. [file elife-85921-fig6-figsupp2-data3.zip › Fig6_SuppFig2_SourceData5/Fig6_SuppFig2D_CTX_Sp1_sourceblot.tif]

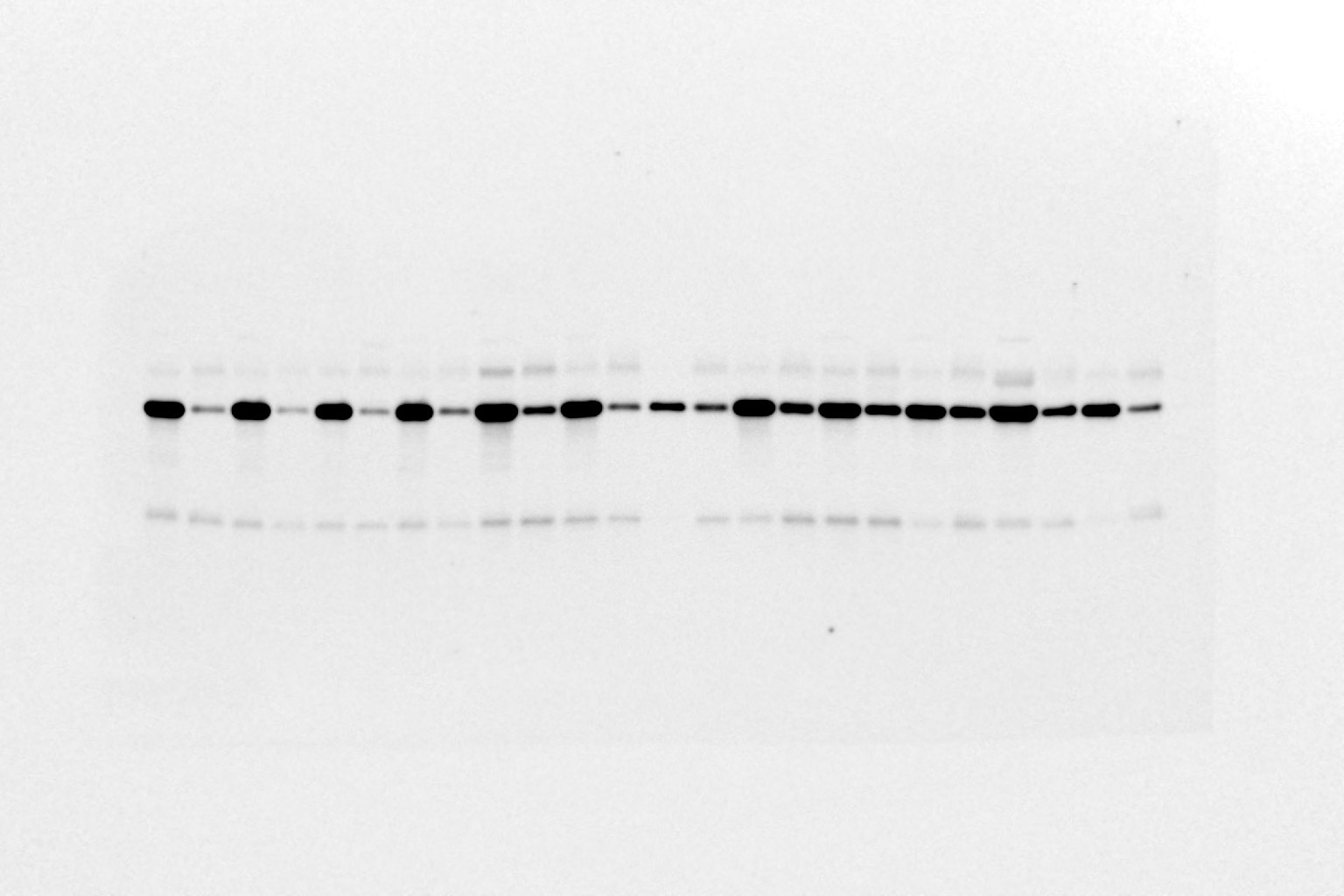

Supplement: Figure 6—figure supplement 2—source data 3. [file elife-85921-fig6-figsupp2-data3.zip › Fig6_SuppFig2_SourceData5/Fig6_SuppFig2D_CTX_TDP43_sourceblot.tif]

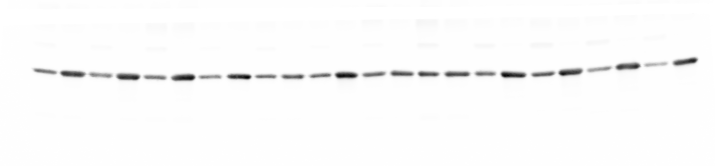

Supplement: Figure 6—figure supplement 2—source data 3. [file elife-85921-fig6-figsupp2-data3.zip › Fig6_SuppFig2_SourceData5/Fig6_SuppFig2G_CTX_GAPDH_sourceblot.tif]

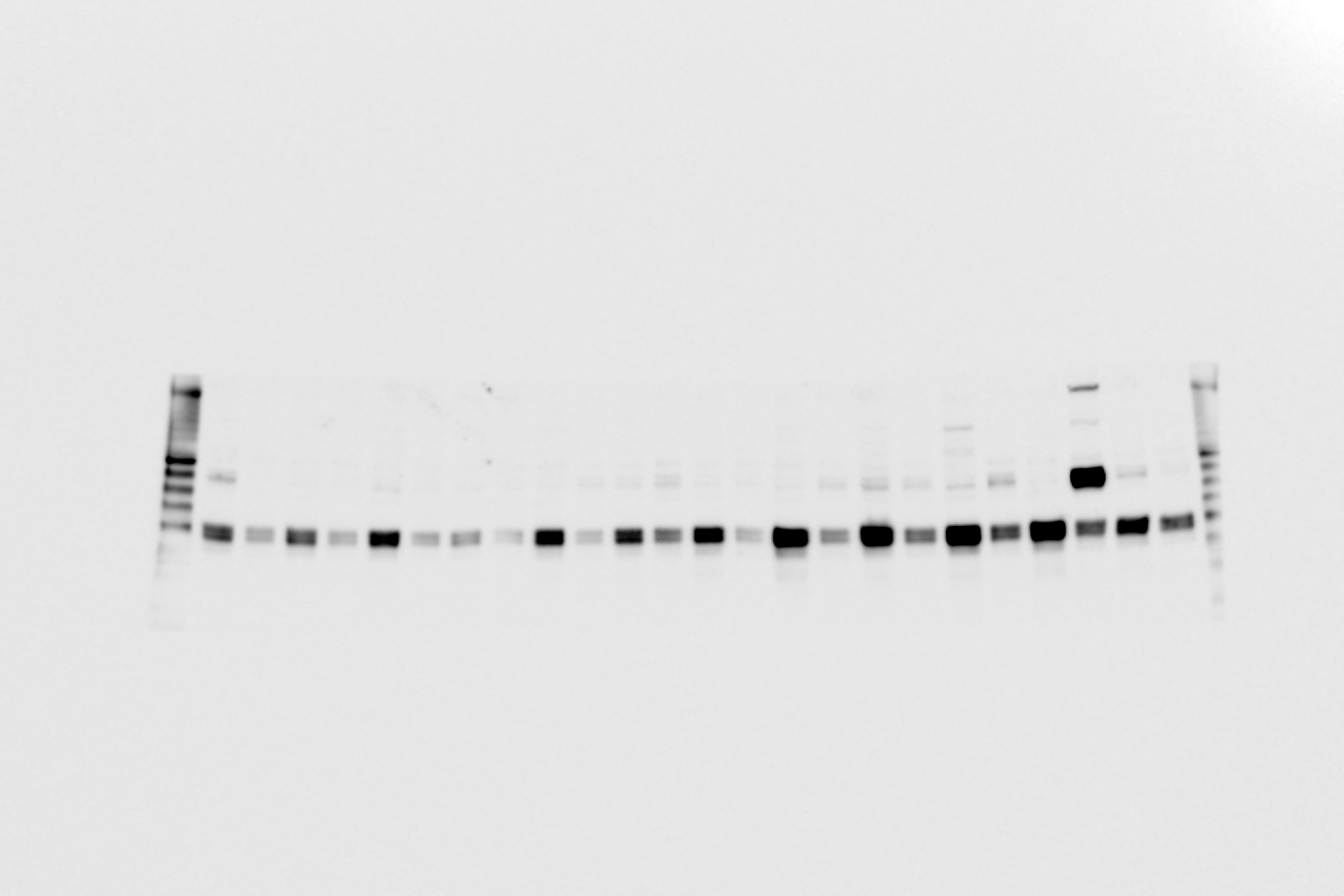

Supplement: Figure 6—figure supplement 2—source data 3. [file elife-85921-fig6-figsupp2-data3.zip › Fig6_SuppFig2_SourceData5/Fig6_SuppFig2G_CTX_Sp1_sourceblot.tif]

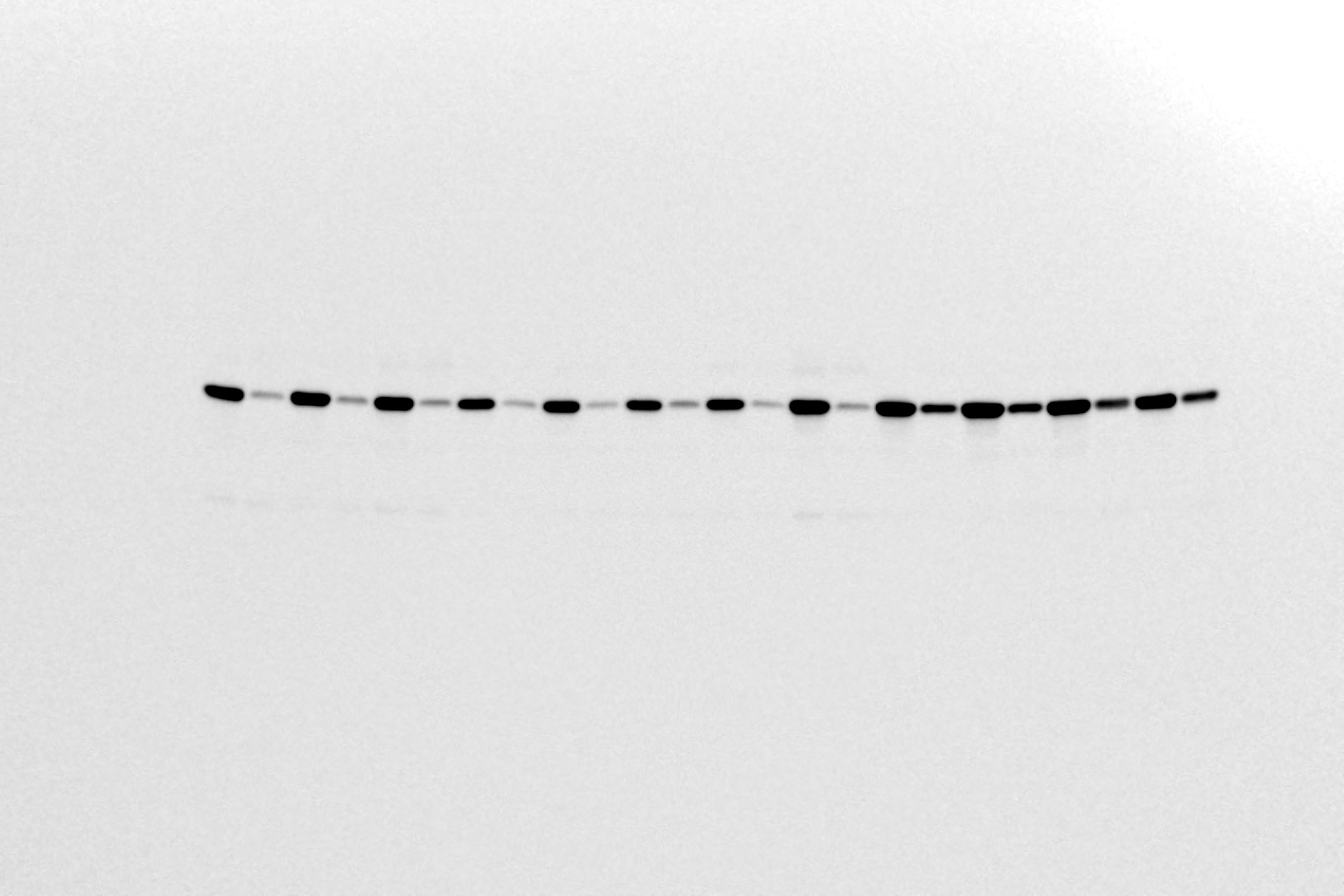

Supplement: Figure 6—figure supplement 2—source data 3. [file elife-85921-fig6-figsupp2-data3.zip › Fig6_SuppFig2_SourceData5/Fig6_SuppFig2G_CTX_TDP43_sourceblot.tif]

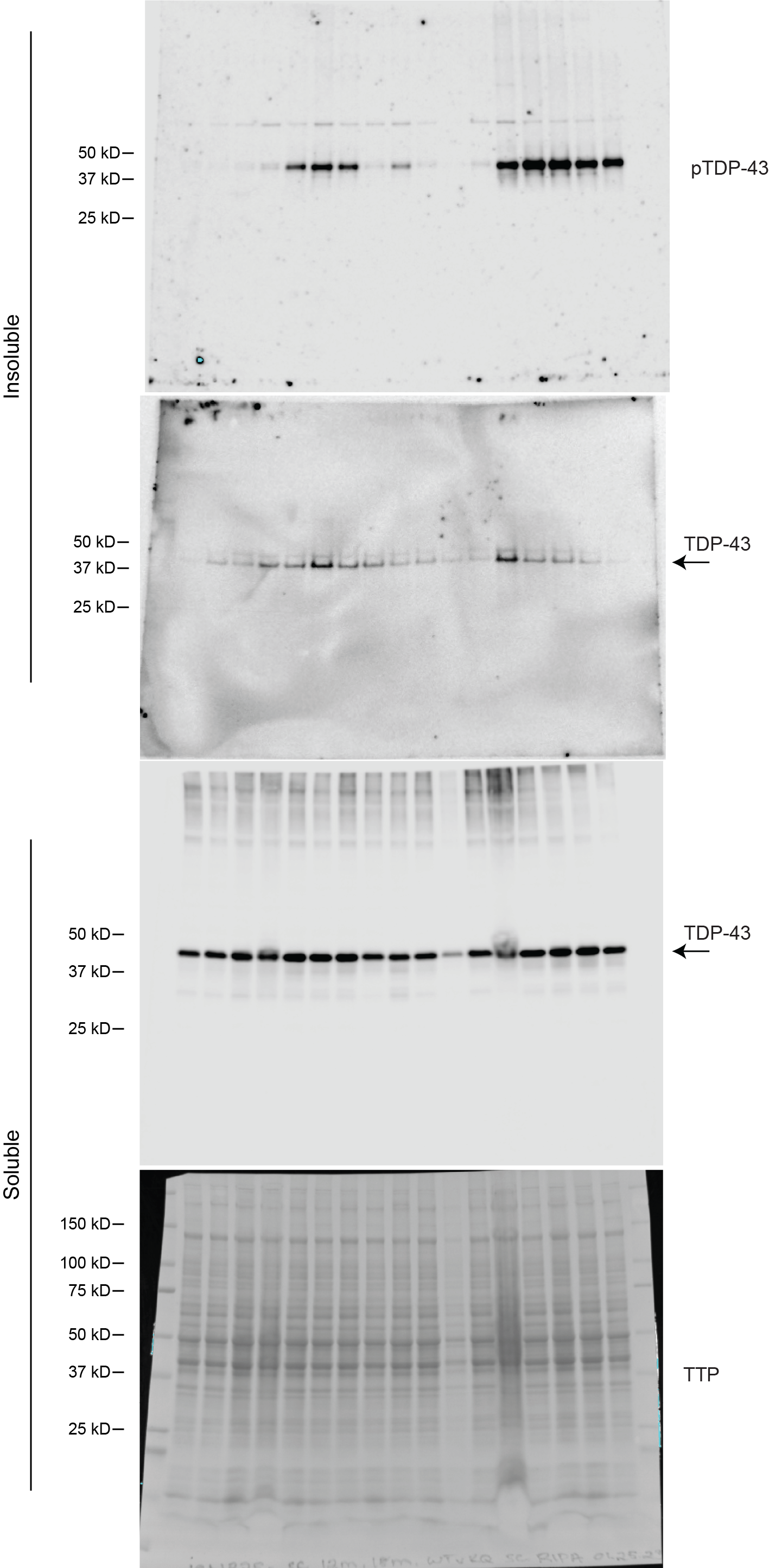

Supplement: Figure 6—figure supplement 3—source data 2. [file elife-85921-fig6-figsupp3-data2.zip › Fig2_SuppFig3_SourceData2/Fig6_SuppFig3_SourceData2_A.png]

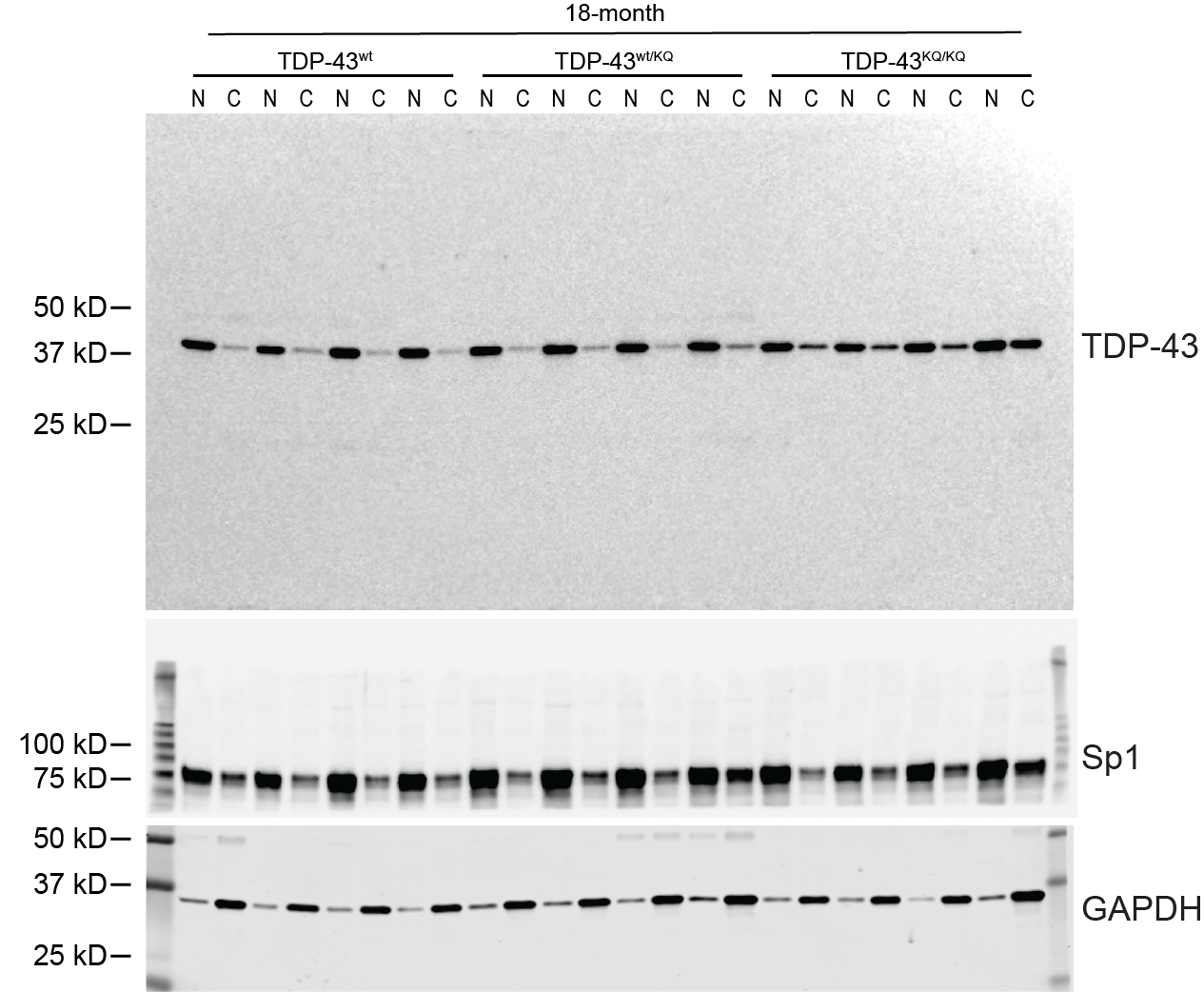

Supplement: Figure 6—figure supplement 3—source data 2. [file elife-85921-fig6-figsupp3-data2.zip › Fig2_SuppFig3_SourceData2/Fig6_SuppFig3_SourceData2_D.png]

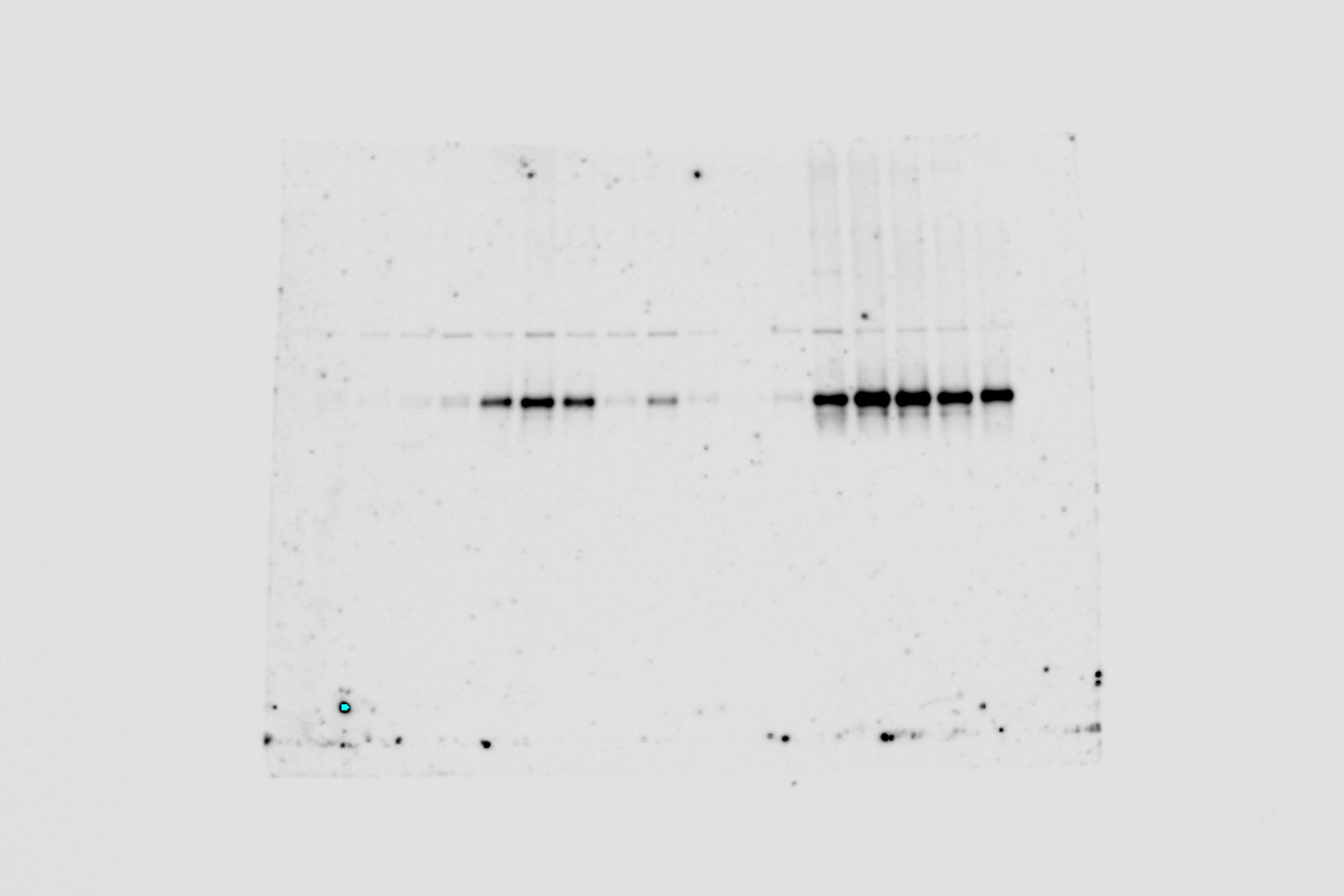

Supplement: Figure 6—figure supplement 3—source data 3. [file elife-85921-fig6-figsupp3-data3.zip › Fig6_SuppFig3_SourceData4/Fig6_SuppFig3A_SC_Insolp409.410TDP43_sourceblot.tif]

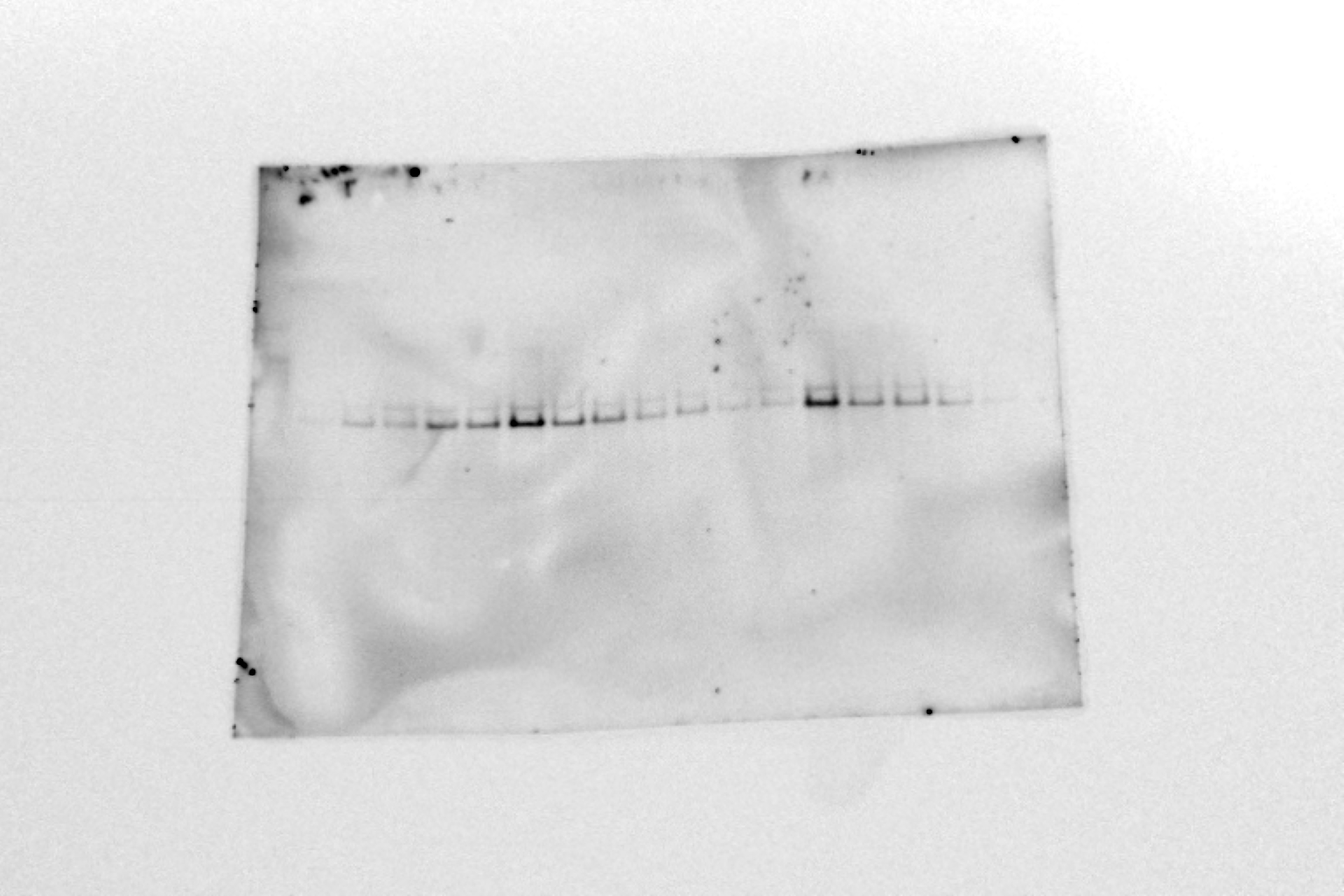

Supplement: Figure 6—figure supplement 3—source data 3. [file elife-85921-fig6-figsupp3-data3.zip › Fig6_SuppFig3_SourceData4/Fig6_SuppFig3A_SC_InsolTDP43_sourceblot.tif]

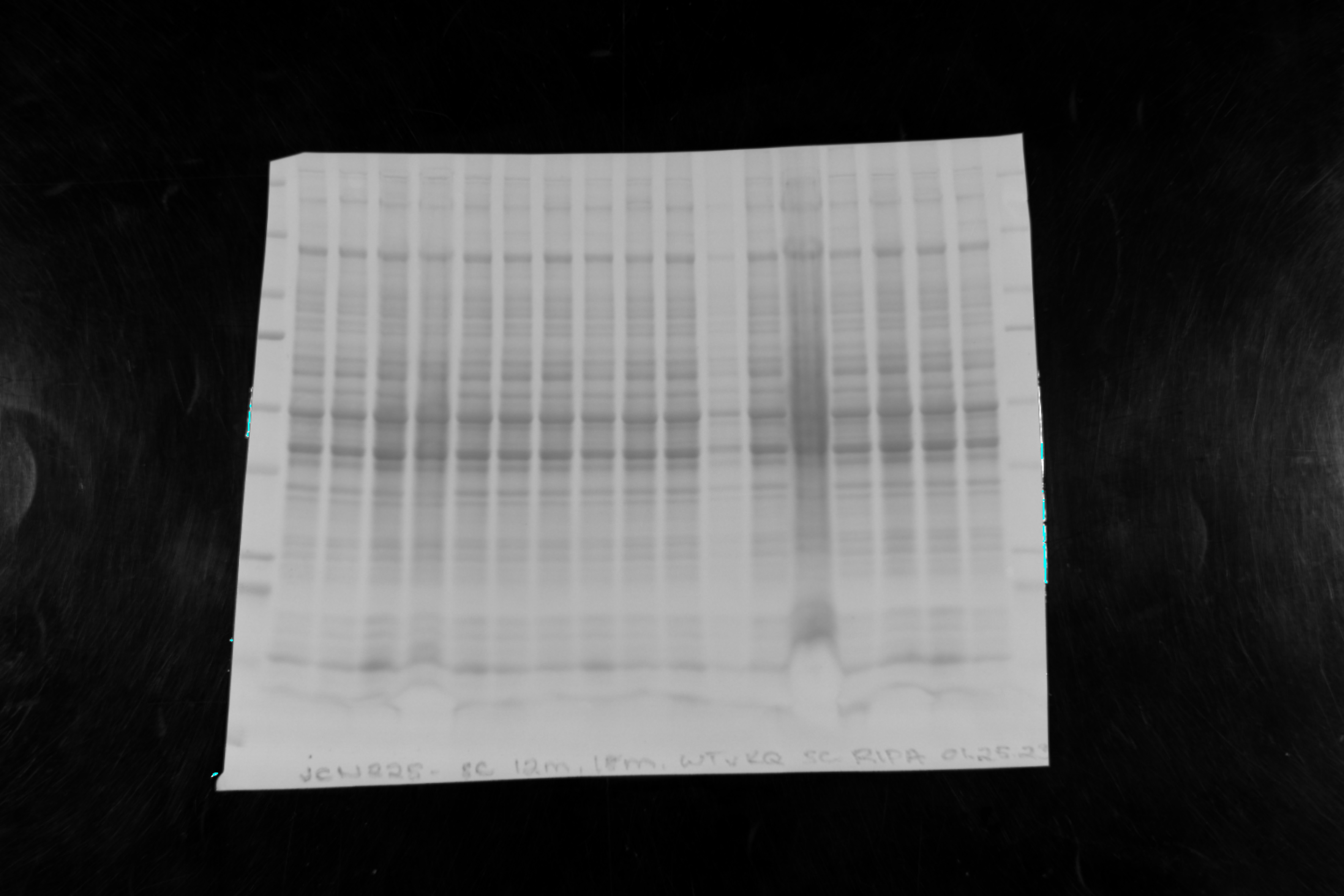

Supplement: Figure 6—figure supplement 3—source data 3. [file elife-85921-fig6-figsupp3-data3.zip › Fig6_SuppFig3_SourceData4/Fig6_SuppFig3A_SC_RIPAsol_Ponceau-TTP_sourceblot.tif]

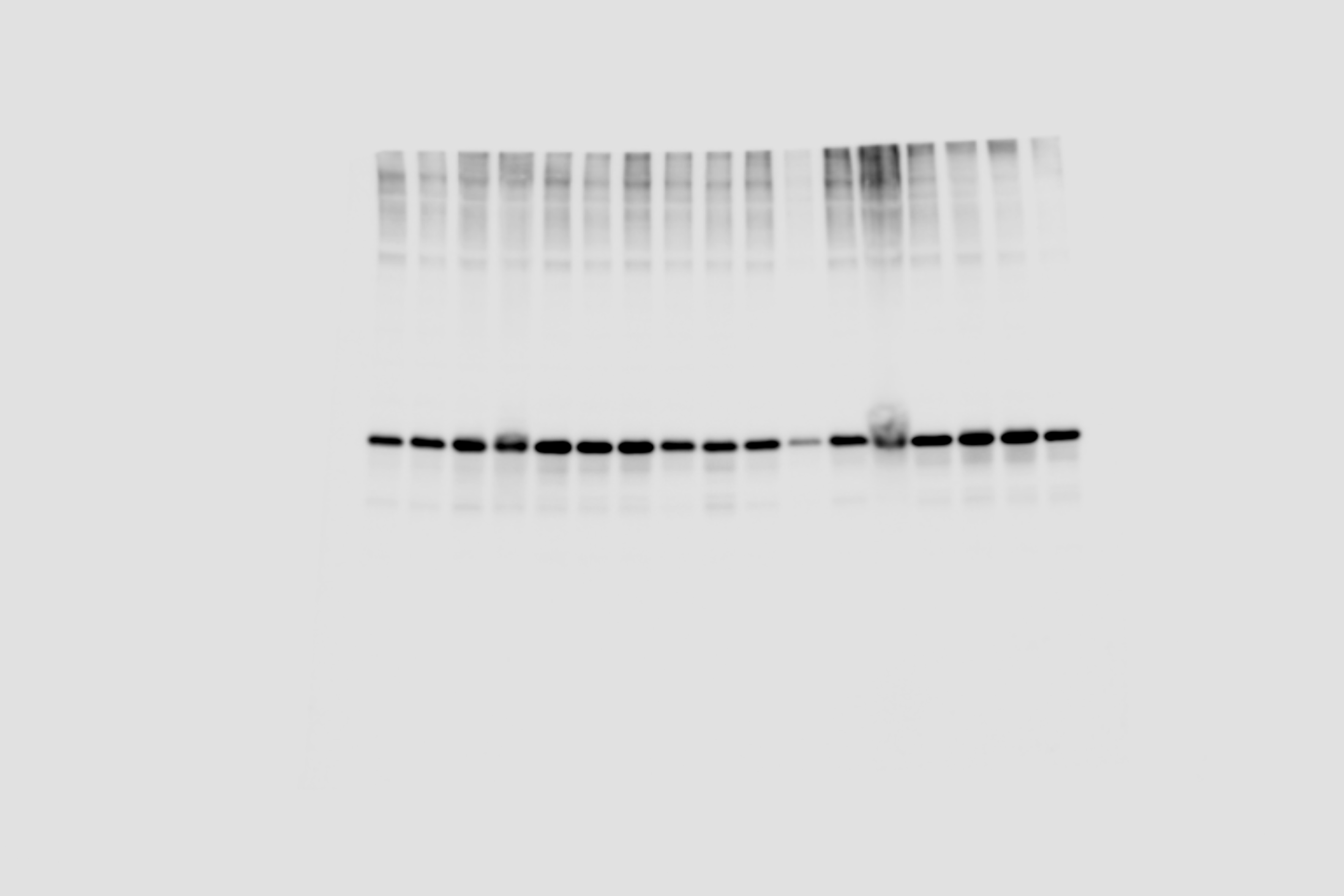

Supplement: Figure 6—figure supplement 3—source data 3. [file elife-85921-fig6-figsupp3-data3.zip › Fig6_SuppFig3_SourceData4/Fig6_SuppFig3A_SC_RIPAsol_TDP43_sourceblot.tif]

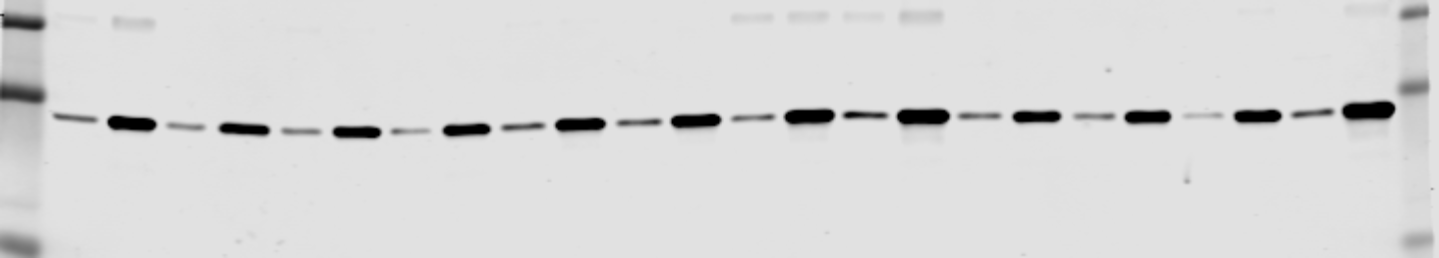

Supplement: Figure 6—figure supplement 3—source data 3. [file elife-85921-fig6-figsupp3-data3.zip › Fig6_SuppFig3_SourceData4/Fig6_SuppFig3D_SC_GAPDH_sourceblot.tif]

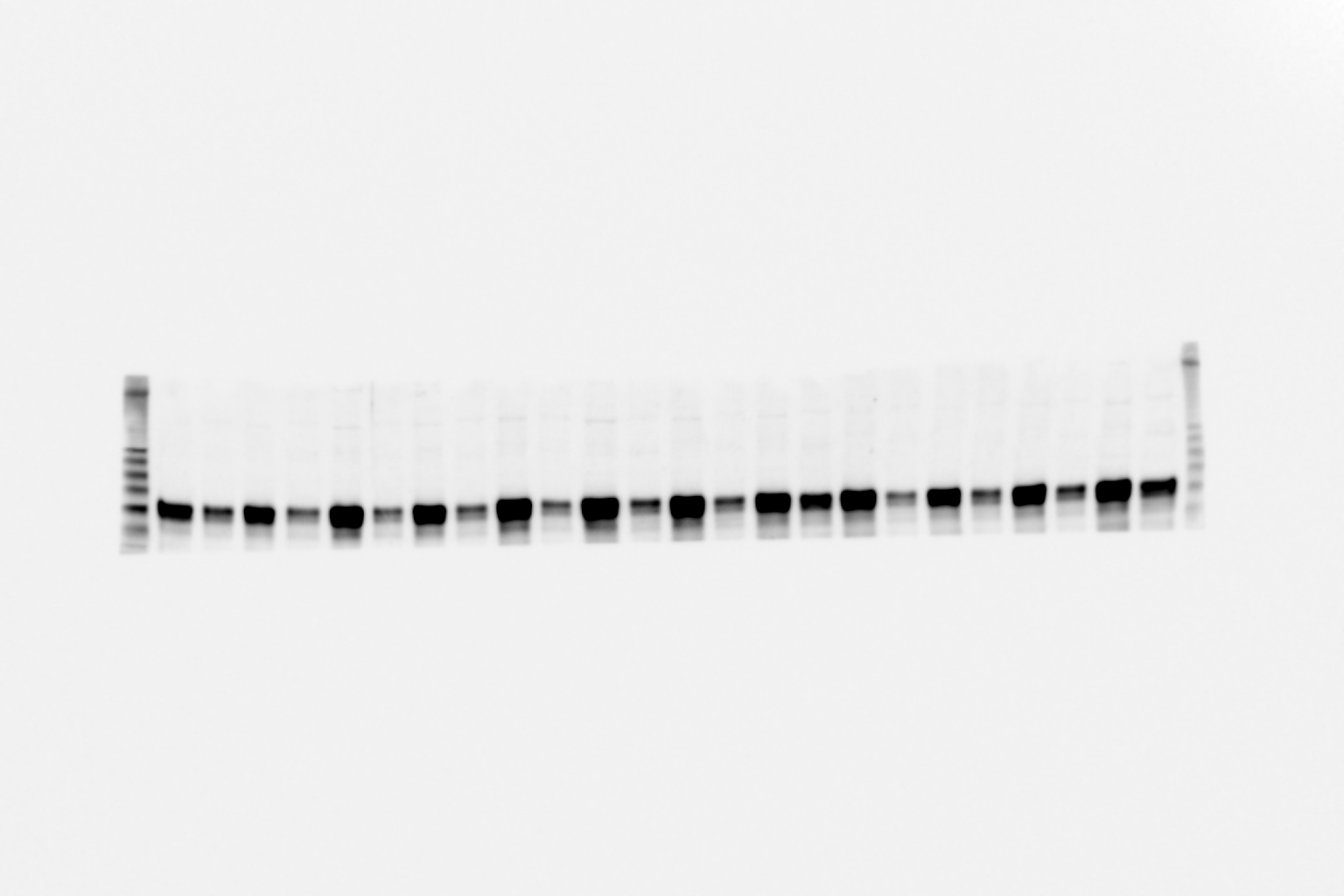

Supplement: Figure 6—figure supplement 3—source data 3. [file elife-85921-fig6-figsupp3-data3.zip › Fig6_SuppFig3_SourceData4/Fig6_SuppFig3D_SC_Sp1_sourceblot.tif]

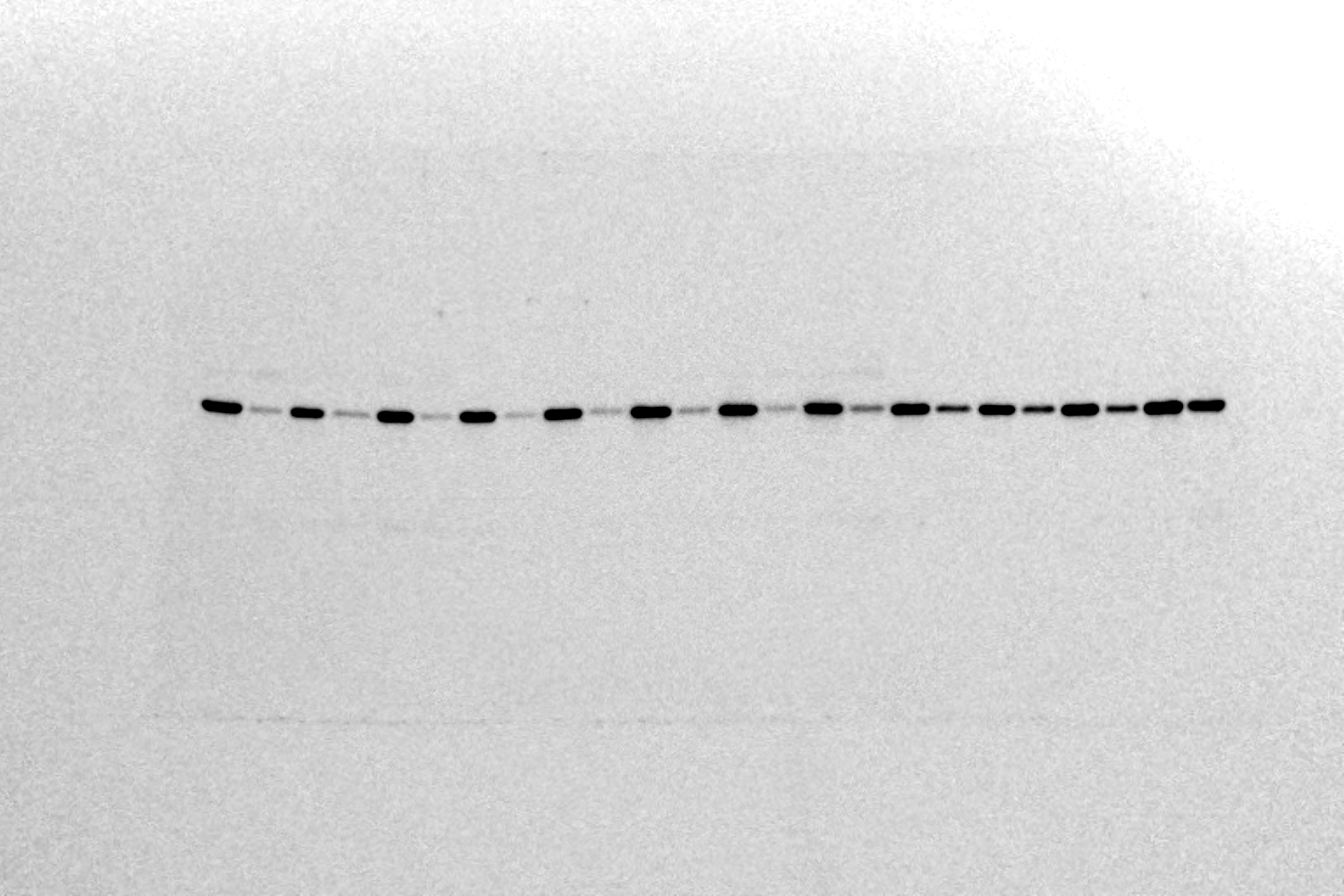

Supplement: Figure 6—figure supplement 3—source data 3. [file elife-85921-fig6-figsupp3-data3.zip › Fig6_SuppFig3_SourceData4/Fig6_SuppFig3D_SC_TDP43_sourceblot.tif]

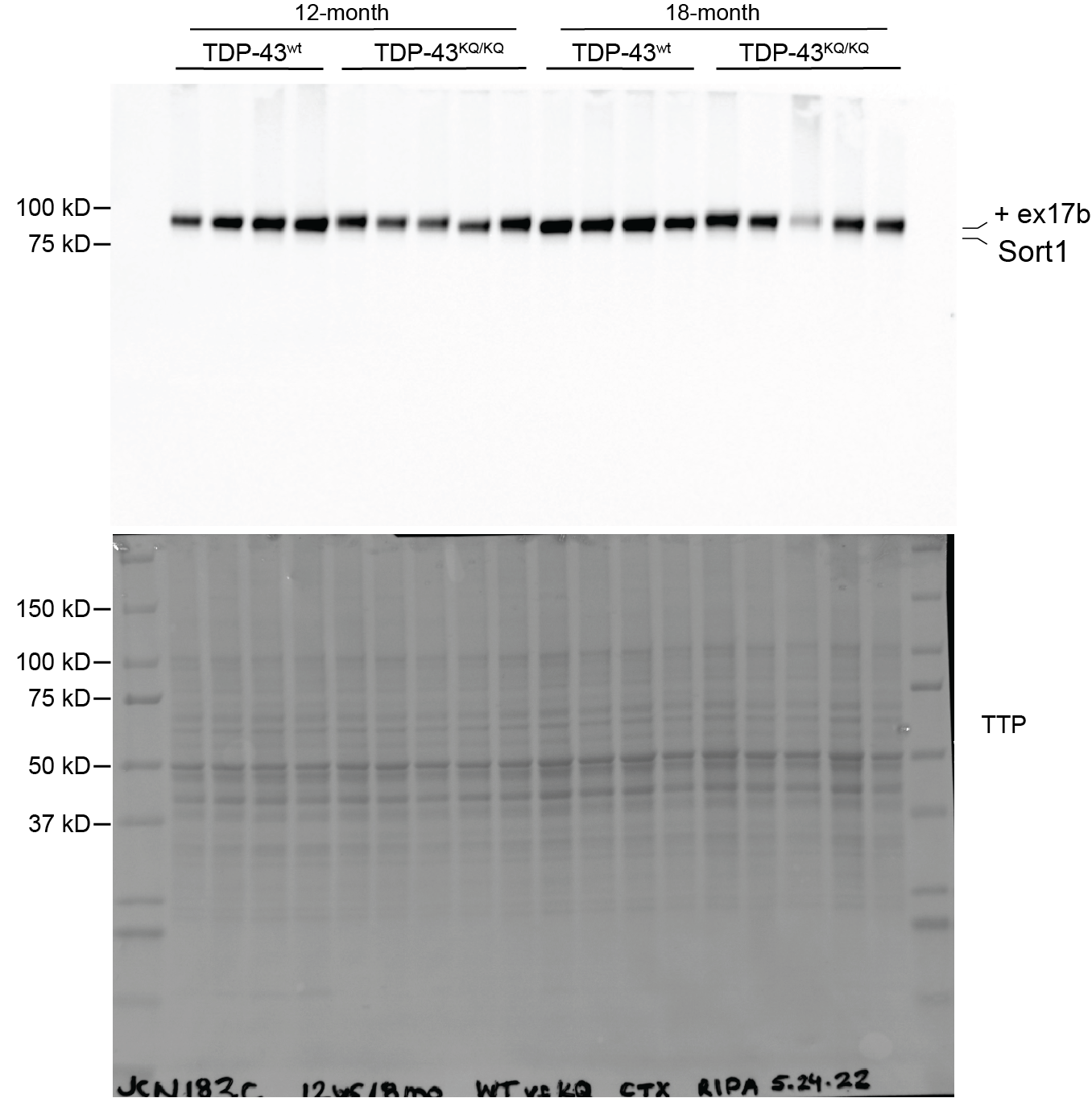

Supplement: Figure 8—source data 2. [file elife-85921-fig8-data2.zip › Fig8_SourceData2/Fig8_SourceData2_A.png]

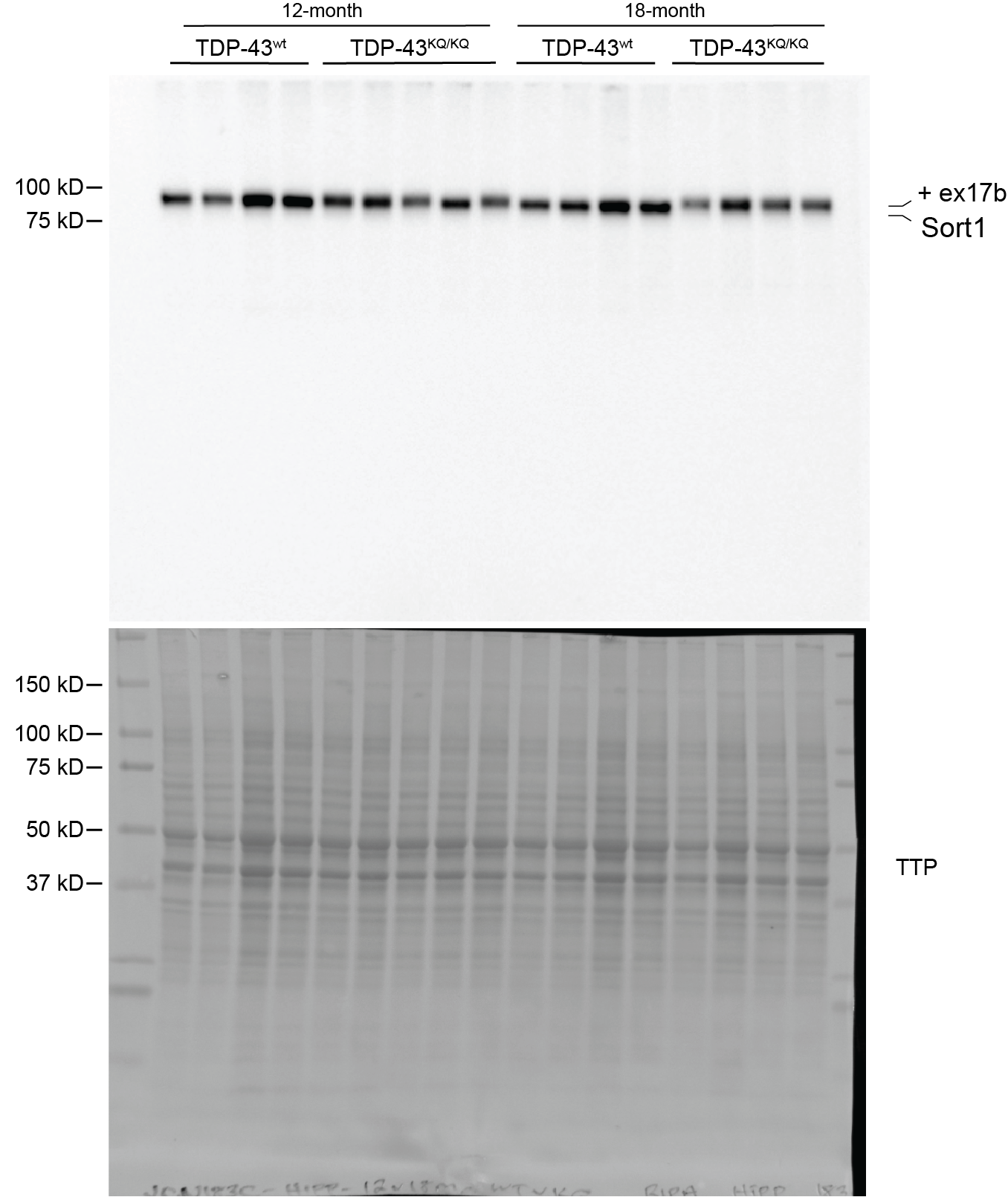

Supplement: Figure 8—source data 2. [file elife-85921-fig8-data2.zip › Fig8_SourceData2/Fig8_SourceData2_D.png]

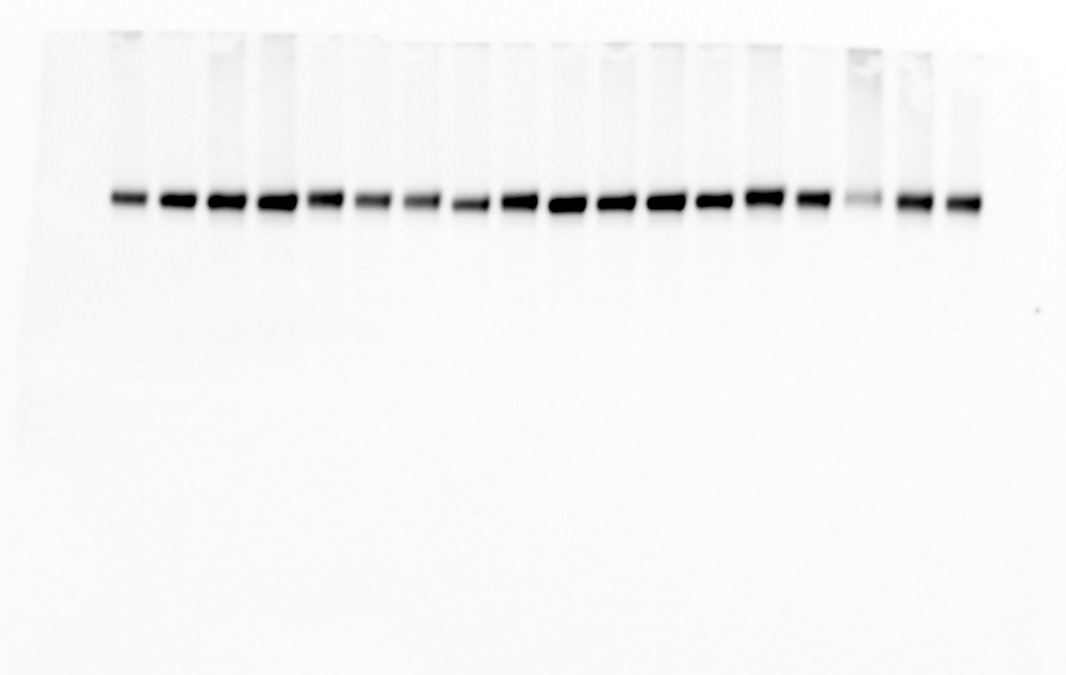

Supplement: Figure 8—source data 3. [file elife-85921-fig8-data3.zip › Fig8_SourceData4/Fig8D_CTX_RIPAsol_SORT1_sourceblot.tif]

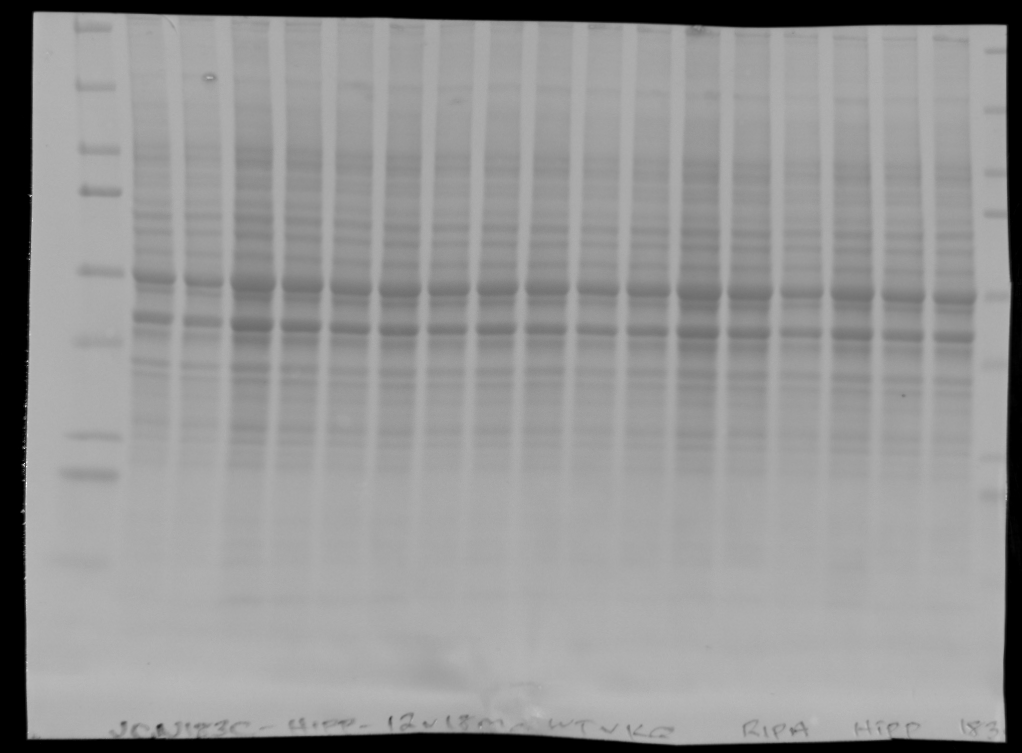

Supplement: Figure 8—source data 3. [file elife-85921-fig8-data3.zip › Fig8_SourceData4/Fig8E_Hipp_RIPAsol_Ponceau-TTP_sourceblot.tif]

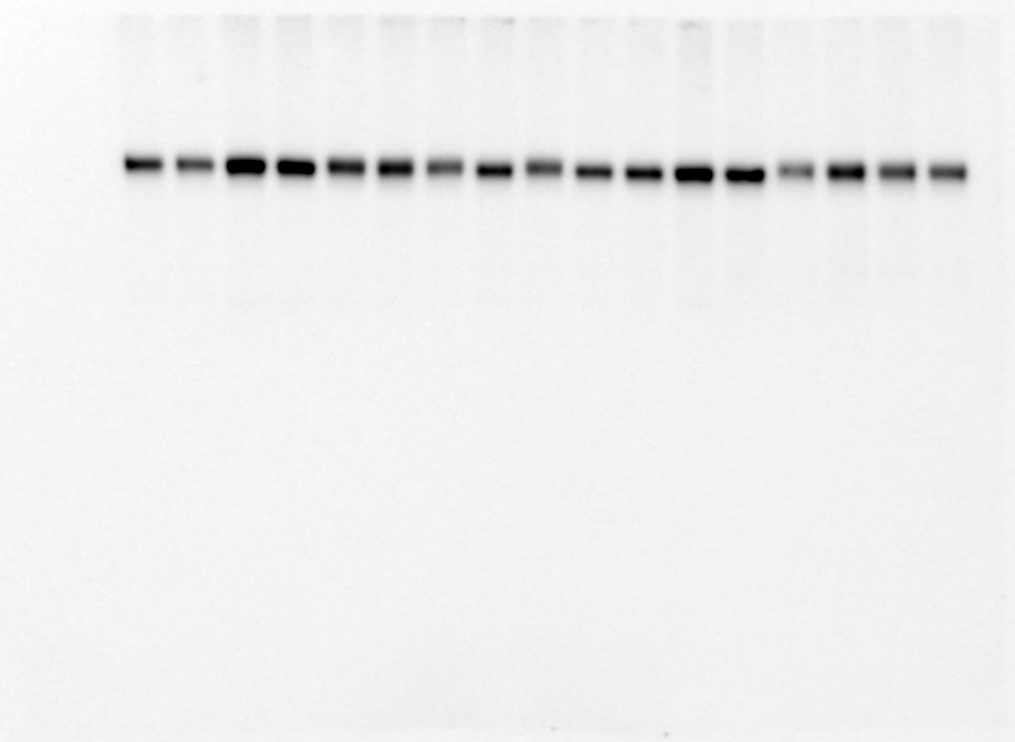

Supplement: Figure 8—source data 3. [file elife-85921-fig8-data3.zip › Fig8_SourceData4/Fig8E_Hipp_RIPAsol_SORT1_sourceblot.tif]
